# Changes in Socioeconomic Disparities for Traffic-Related Air Pollution Exposure During Pregnancy Over a 20-Year Period in Texas

**DOI:** 10.1001/jamanetworkopen.2023.28012

**Published:** 2023-08-11

**Authors:** Mary D. Willis, Elaine L. Hill, Collette N. Ncube, Erin J. Campbell, Lena Harris, Max Harleman, Beate Ritz, Perry Hystad

**Affiliations:** 1 Department of Epidemiology, School of Public Health, Boston University, Boston, Massachusetts; 2 Department of Public Health Sciences, School of Medicine and Dentistry, University of Rochester, Rochester, New York; 3 Department of Government and Sociology, College of Arts and Sciences, Georgia College and State University, Milledgeville; 4 Department of Epidemiology, Fielding School of Public Health, University of California, Los Angeles; 5 School of Biological and Population Health Sciences, College of Public Health and Human Sciences, Oregon State University, Corvallis

## Abstract

**Importance:**

Air pollution presents clear environmental justice issues. However, few studies have specifically examined traffic-related air pollution (TRAP), a source driven by historically racist infrastructure policies, among pregnant individuals, a population susceptible to air pollution effects. How these disparities have changed over time is also unclear but has important policy implications.

**Objective:**

To examine changes in TRAP exposure by sociodemographic characteristics among recorded pregnancies over a 20-year period.

**Design, Setting, and Participants:**

This population-based birth cohort study used descriptive analysis among pregnant individuals in Texas from 1996 to 2016. All pregnant individuals with valid residential address, socioeconomic, and demographic data were included. Individual-level race and ethnicity, education, and maternal birthplace data were extracted from birth certificates and neighborhood-level household income and historical neighborhood disinvestment (ie, redlining) data were assessed via residential addresses. Data analysis occurred between June 2022 and June 2023.

**Main Outcomes and Measures:**

The main outcome, TRAP exposure at residential addresses, was assessed via traffic levels, represented by total and truck-specific vehicle miles traveled (VMT) within 500 m; nitrogen dioxide (no_2_) concentrations from a spatial-temporal land use regression model (ie, vehicle tailpipe emissions); and National Air Toxic Agency cancer risk index from on-road vehicle emissions. TRAP exposure differences were assessed by sociodemographic indicators over the 1996 to 2016 period.

**Results:**

Among 7 043 598 pregnant people (mean [SD] maternal age, 26.8 [6.1] years) in Texas from 1996 to 2016, 48% identified as Hispanic or Latinx, 4% identified as non-Hispanic Asian or Pacific Islander, 12% identified as non-Hispanic Black, and 36% identified as non-Hispanic White. There were differences in TRAP for pregnant people by all sociodemographic variables examined. The absolute level of these disparities decreased from 1996 to 2016, but the relative level of these disparities increased: for example, in 1996, non-Hispanic Black pregnant individuals were exposed to a mean (SD) 15.3 (4.1) ppb of no_2_ vs 13.5 (4.4) ppb of no_2_ for non-Hispanic White pregnant individuals, compared with 2016 levels of 6.7 (2.4) ppb no_2_ for Black pregnant individuals and 5.2 (2.4) ppb of no_2_ for White pregnant individuals. Large absolute and relative differences in traffic levels were observed for all sociodemographic characteristics, increasing over time. For example, non-Hispanic Black pregnant individuals were exposed to a mean (SD) of 22 836 (32 844) VMT within 500 m of their homes, compared with 12 478 (22 870) VMT within 500 m of the homes of non-Hispanic White pregnant individuals in 2016, a difference of 83%.

**Conclusions and Relevance:**

This birth cohort study found that while levels of air pollution disparities decreased in absolute terms over the 20 years of the study, relative disparities persisted and large differences in traffic levels remained, requiring renewed policy attention.

## Key Points

**Question:**

What are the environmental justice implications of traffic-related air pollution exposure for pregnant people and how have exposure disparities changed over time?

**Findings:**

In this birth cohort study including 7 043 598 pregnant people from 1996 to 2016 in Texas, traffic-related air pollution exposure levels decreased substantially for all pregnant people, likely due to reduced tailpipe emissions, but relative disparities for persistently marginalized groups remained. Much higher traffic levels, which increased over time, were observed for persistently marginalized groups.

**Meaning:**

These findings suggest that regulations successfully reduced tailpipe emission exposures, but traffic air pollution (and other environmental exposures associated with traffic) remains a critical environmental justice issue.

## Teaser

This cohort study examines changes in traffic-related air pollution among pregnant people by sociodemographic characteristics in Texas from 1996 to 2016.

## Introduction

Air pollution is associated with more than 100 000 deaths per year in the United States, as well as adverse health outcomes (eg, preterm birth, asthma exacerbations, and heart disease).^1,2,3,4,5^ Substantial disparities in air pollution exposure and health effects exist across socioeconomic and racial and ethnic gradients.^6,7,8,9,10,11,12,13,14,15^ While decades of environmental policy have improved air quality across the United States,^16,17,18^ these improvements may not offset disparities in exposure and associated health outcomes if regulations disproportionately benefit White and high-income communities.^9,10,11,12,19,20,21^

Traffic-related air pollution (TRAP) may be a particularly important source of air pollution disparities for environmental justice.^22^ Historical, racist infrastructure policies resulted in the disproportionate placement of highways in Black and Brown, low income, and disadvantaged communities,^23^ yielding higher concentrations of TRAP.^9,10,11^ TRAP is a toxic subset of ambient air pollution, including byproducts of fossil fuel combustion, road dust, and brake wear.^24^ This type of pollution disperses along highly localized gradients, largely concentrated within 500 m of major roads.^15,25,26,27^ TRAP is amenable to policies for exposure reductions, such as regulations to reduce tailpipe emissions or public investment in sound and pollution barriers. How these types of changes have impacted environmental justice patterns over time is largely unknown, especially for susceptible populations and disadvantaged communities.

Pregnancy is a vulnerable time period during which air pollution may have particularly deleterious effects.^28,29^ TRAP has been associated with adverse pregnancy outcomes (eg, infertility, spontaneous abortion, gestational hypertension, preterm birth, intrauterine growth restriction).^28,29,30,31,32,33,34^ Risks of severe pregnancy complications are higher among Hispanic or Latinx, non-Hispanic Black, and lower-income people.^35,36^ Therefore, environmental risk factors with strong socioeconomic and racial and ethnic gradients, such as TRAP, can simultaneously exacerbate preexisting health disparities among persistently marginalized communities.

In this study, we leveraged data from vital statistics birth records in Texas, a state that has spent billions of dollars on TRAP reductions and highway infrastructure. We evaluated the differences among residential exposures by sociodemographic gradients via detailed measures of total and truck-specific vehicle miles traveled (VMT), nitrogen dioxide (no_2_), and a cancer risk index from on-road vehicle emissions. We further quantified how the magnitude of exposure disparity changed over time between the most and least advantaged groups (defined by characteristics such as race, ethnicity, and educational attainment). We hypothesized that the absolute and relative disparity based on TRAP markers would be reduced among all recorded pregnancies from 1996 to 2016.

## Methods

This birth cohort study was approved by the institutional review board at Oregon State University and the Texas Department of State Health Services with a waiver of the requirement for informed consent. The study was based on secondary data, so no individuals were contacted and it posed no more than minimal risk to the individuals included, did not adversely affect their rights, and had no effect on their welfare. We used the Strengthening the Reporting of Observational Studies in Epidemiology (STROBE) reporting guideline for cohort studies.

### Study Population

We leveraged birth certificate data from the Texas Department of Health and Human Services to identify all recorded pregnancies in Texas from 1996 to 2016. We excluded records without residential address data and records with missing or invalid socioeconomic and demographic characteristics.

### Measures of Sociodemographic Characteristics

We used data from the birth certificates to measure sociodemographic characteristics of pregnant individuals. Due to changes related to data collected on birth certificates over time, we examined variables that have been collected consistently from 1996 to 2016: maternal race and ethnicity (Hispanic or Latinx, non-Hispanic Asian or Pacific Islander, non-Hispanic Black, or non-Hispanic White); maternal educational attainment (completed high school, education beyond high school), and maternal birthplace (US-born, non–US-born). Data changes precluded straightforward analysis of other socioeconomic characteristics (eg, insurance type, Special Supplemental Nutrition Program for Women, Infants, and Children eligibility).

We also linked 2 neighborhood-level characteristics to each residential address: median household income levels and historical neighborhood disinvestment. Census tract data from the American Community Survey were used for 2007 to 2016 births, and we applied linear interpolation models on the census tract level Decennial Census and American Community Survey data for births from 1996 to 2006. We created tertiles of household income by year to standardize median household income over time. We measured historical neighborhood disinvestment via Home Owners’ Loan Corporation (HOLC) grades,^37^ which, starting in 1933, assigned mortgage security risks to specific neighborhoods and inhibited economic gains in areas with high concentrations of racially minoritized people (ie, redlining).^38,39^ For the 7% of births within a neighborhood region in the HOLC database, we assigned the corresponding mortgage security grade (A-D), in which A was considered least risky and D, most risky.

### TRAP Exposures

We used 3 methods to estimate exposure to TRAP for the residential addresses reported at delivery. First, we used a historical statewide database of traffic volumes from the Texas Roadway Inventory data.^40^ Annual Average Daily Traffic (AADT; calculated as total traffic volume in a year divided by 365 days) was available from the 1999 to 2016 study period, supplemented with linear extrapolation models to estimate AADT for 1996 to 1998. We calculated VMT and truck-specific VMT exposures (via Texas Department of Transportation calculations) for all roads within 500 m of the residential addresses by multiplying the AADT on a given road segment by its length within the buffer and adding this product across all roads within the buffer (ie, total VMT within 500 m). Second, we assigned annual no_2_ concentrations in parts per billion (ppb) from 1996 to 2016 using an existing hybrid model estimate.^41,42^ Third, we assigned a census tract–level index of total cancer risk from on-road vehicle emissions from the National Air Toxics Assessment (NATA),^43^ where inventories for 1999 were assigned to 1996 to 2000 births, 2002 inventories to 2001 to 2003 births, 2005 inventories to 2004 to 2008 births, 2011 inventories to 2009 to 2012 births, and 2014 inventories to 2013 to 2016 births. While NATA methodology has changed substantially over time, we focused on within-year differences in exposure disparities, thus preventing inaccurate cross-year comparisons.

### Statistical Analysis

We present descriptive statistics of sociodemographic characteristics for pregnant people from 1996 to 2016 along with changes in VMT at 500 m, truck VMT at 500 m, no_2_, and the NATA on-road cancer risk estimates. We visually examine disparities in these TRAP exposures by sociodemographic characteristics from 1996 to 2016 and summarize exposure levels, for which we used the group with the highest socioeconomic positioning as the reference (ie, non-Hispanic White individuals for race and ethnicity, completed more than high school for education, US-born for place of birth, high-income neighborhood for neighborhood-level income, and A- or B-graded neighborhood for HOLC neighborhood grade). For each group, we computed absolute differences in exposure (via subtraction) and relative differences in exposure (via percentage change). We further disentangle the association of TRAP with individual and neighborhood characteristics by stratifying our individual-level descriptive statistics by neighborhood attributes (eg, neighborhood median household income, historic neighborhood disinvestment). Finally, we examined geographic differences by counties and census tracts by mapping exposure differences by sociodemographic characteristics at these geographic scales.

Statistical analyses were conducted using Stata version 16.1 (StataCorp). Data analysis occurred between June 2022 and June 2023.

## Results

Of 8 114 440 births recorded in Texas from 1996 to 2016, 890 842 records (11.0%) without residential address data and 180 000 records (2.2%) with missing or invalid socioeconomic and demographic characteristics were excluded. In total, these criteria yielded 7 043 598 birth records (mean [SD] maternal age, 26.8 [6.1] years) for analysis. We first examined sociodemographic and TRAP characteristics of pregnant people in Texas, focusing on changes over time between 1996 and 2016 (Table 1). The cohort included 48% Hispanic or Latinx individuals, 4% non-Hispanic Asian or Pacific Islander individuals, 12% non-Hispanic Black individuals, and 36% White non-Hispanic individuals; 29% of individuals were born outside the US, and the census tract mean (SD) household income was $46 099 ($27 757).

**Table 1.** Sociodemographic Characteristics of Individuals Delivering Live-Born Infants in Texas, 1996 to 2016

Characteristic	Individuals, No. (%)		
	1996 (n = 273 527)	2006 (n = 352 473)	2016 (n = 382 425)
Race and ethnicity			
Hispanic or Latinx	116 932 (43)	171 364 (49)	180 600 (47)
Non-Hispanic Asian or Pacific Islander	8093 (3)	13 340 (4)	21 676 (6)
Non-Hispanic Black	33 758 (12)	42 506 (12)	48 014 (13)
Non-Hispanic White	114 074 (42)	123 535 (35)	130 756 (34)
Non-Hispanic other^a^	670 (<1)	1728 (<1)	1379 (<1)
≤High school education	173 443 (63)	197 440 (56)	172 024 (45)
Non–US-born	70 421 (26)	105 230 (30)	107 319 (28)
Median household income, mean (SD), $^b^	32 640 (27 813)	45 452 (25 478)	57 853 (28 679)
VMT 500 m, mean (SD)			
Overall	17 086 (24 731)	15 751 (24 385)	16 874 (27 482)
Truck-specific	922 (1721)	876 (1731)	971 (1967)
NATA vehicle cancer risk, mean (SD)	7.2 (3.6)	6.7 (3.6)	2.9 (1.4)
no_2_, mean (SD), ppb	14.7 (4.7)	10.3 (3.6)	6.1 (2.6)
HOLC ^c^			
A or B	9461	8654	7380
C or D	17 409	15 589	12 630

Abbreviations: HOLC, Home Owners’ Loan Corporation; NATA, National Air Toxics Assessment; no_2_, nitrogen dioxide; VMT, vehicle miles traveled.

^a^ Non-Hispanic other encompasses responses that were too small for group-level analysis (eg, American Indian) and other options that did not align with the previous categories.

^b^ Data are from the US Census at the census tract level.

^c^ Data are from the Home Owners’ Loan Corporation (HOLC, est. 1933) grades. Only a subset of the individuals fall into a polygon in the HOLC maps, which is why the data are presented as totals. A or B were classified as the least risky (ie, not redlined) areas; C or D, most risky (ie, redlined) areas.

We observed changes in the distribution of live births across racial and ethnic groups. For instance, 114 074 of 273 527 live births (42%) were to non-Hispanic White individuals in 1996, with a decrease to 130 756 of 382 425 live births (34%) by 2016, while 116 932 live births (43%) were to Hispanic or Latinx individuals in 1996, with an increase to 180 600 live births (47%) by 2016. Concurrently, the proportion of live births to non-Hispanic Black individuals remained stable over the 20-year period (12%-13%). The proportion of pregnancies among people who had attained a high school diploma or less at the time of birth decreased as well, dropping from 173 443 pregnancies (63%) in 1996 down to 172 024 pregnancies (45%) in 2016. The proportion of non–US-born pregnant people remained somewhat stable (26%-30%), and mean (SD) household incomes increased from $32 640 ($27 813) in 1996 to $57 853 ($28 679) in 2016. For VMT within 500 m of the residence, we observed a marginal decrease in overall VMT, starting at a mean (SD) of 17 086 (24 731) VMT in 1996 and decreasing to 16 874 (27 482) VMT in 2016. Truck VMT remained relatively stable over the study period. NATA cancer risk scores declined, largely due to methodological changes, from a mean (SD) of 7.2 (3.6) in 1996 to 2.9 (1.4) in 2016. Outdoor no_2_ concentrations due to local TRAP substantially decreased from 14.7 (4.7) ppb in 1996 to 6.1 (2.6) ppb in 2016.

**Figure 1.**
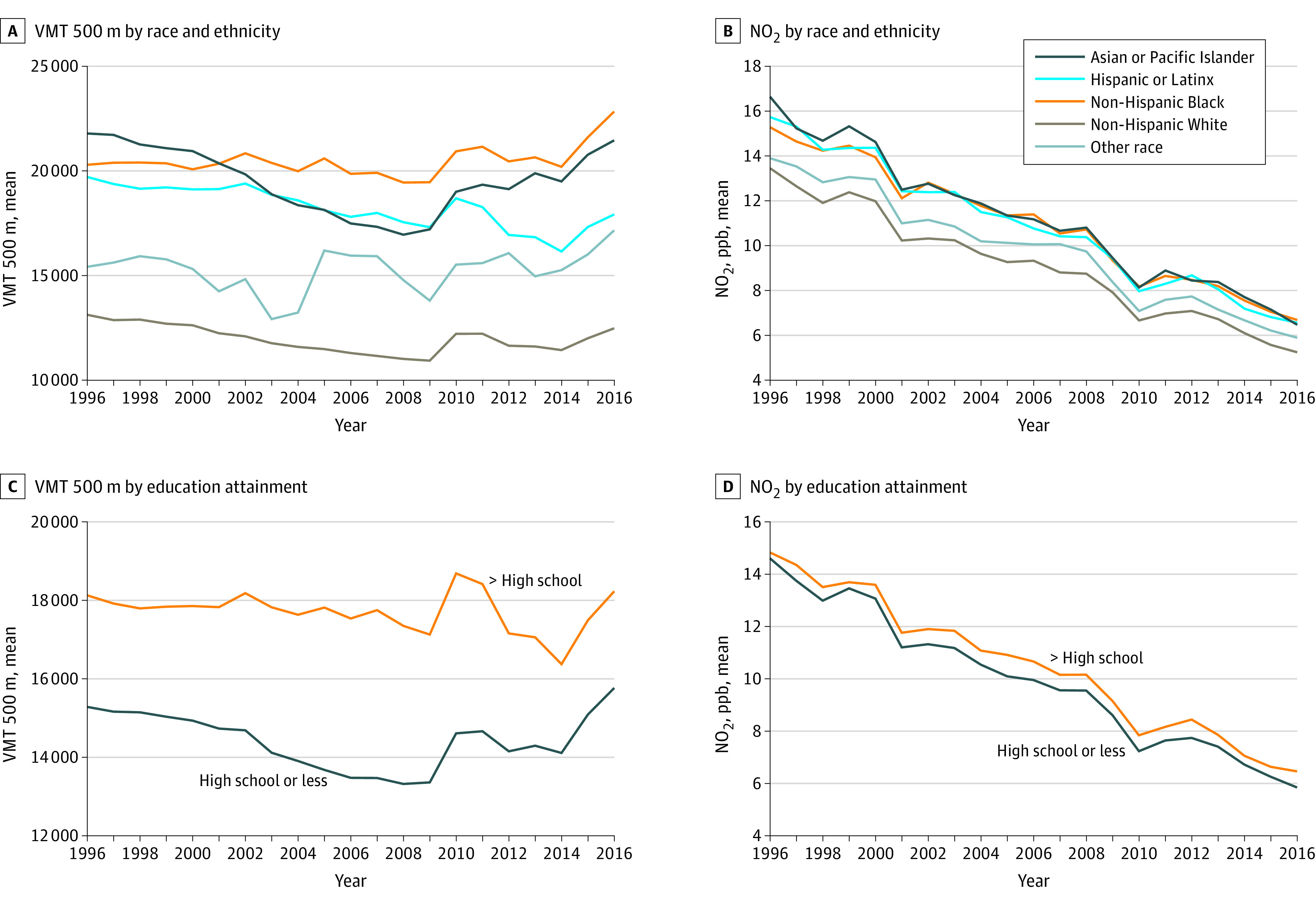
Summary of Nitrogen Dioxide (no_2_) and Vehicle Miles Traveled (VMT) Within 500 m Exposures by Individual Race and Ethnicity and Educational Attainment, 1996-2016 Ppb indicates parts per billion.

When we investigated the magnitude of exposure difference by sociodemographic characteristics, we observed that pregnant individuals with the highest socioeconomic positioning (eg, non-Hispanic White, completed more than high school, US-born, high-income neighborhood) were consistently exposed to less TRAP across all exposure metrics relative to those with lower socioeconomic positioning. Table 2 summarizes measures for 1996 and 2016. For instance, in 1996 and compared with non-Hispanic White individuals, VMT within 500 m of the residential address was 50% higher among Hispanic or Latinx individuals (mean [SD], 13 125 [19 819] VMT vs 19 710 [26 973] VMT), 66% higher among non-Hispanic Asian or Pacific Islander individuals (mean [SD], 21 788 [27 861] VMT), and 55% higher among non-Hispanic Black individuals (mean [SD], 20 293 [28 640] VMT). By 2016 and compared with non-Hispanic White individuals, VMT within 500 m of the residential address was 44% higher among Hispanic or Latinx individuals (mean [SD], 12 478 [22 870] vs 17 919 [27 998] VMT), 72% higher among non-Hispanic Asian or Pacific Islander individuals (mean [SD], 21 460 [31 175] VMT), and 83% higher among non-Hispanic Black individuals(mean [SD], 22 836 [32 844] VMT). VMT and no_2_ exposures by race and ethnicity and educational attainment showed similar trends of higher TRAP exposures for individuals with a high school education or less (Figure 1). These trends were similar for other socioeconomic and demographic characteristics (eFigure 1 in Supplement 1). Results followed this pattern for the other TRAP exposure metrics, with magnitudes larger for truck VMT and lower for no_2_ and NATA exposures (eFigure 2 and eFigure 3 in Supplement 1).

**Table 2.** Summary of Traffic Air Pollution Exposure Differences by Sociodemographic Characteristics for 1996 and 2016

Characteristic	VMT 500 m		Truck VMT 500 m		no_2_ air pollution, ppb		NATA Vehicle Cancer Risk Index	
	Mean (SD)	Difference, estimate (%)	Mean (SD)	Difference, estimate (%)	Mean (SD)	Difference, estimate (%)	Mean (SD)	Difference, estimate (%)
**1996**								
Race and ethnicity								
Hispanic or Latinx	19 710 (26 973)	6585 (50)	1085 (1908)	387 (55)	15.7 (4.8)	2.2 (17)	7.8 (3.7)	1.4 (22)
Non-Hispanic Asian or Pacific Islander	21 788 (27 861)	8663 (66)	963 (1704)	265 (38)	16.6 (3.9)	3.1 (24)	7.9 (2.7)	1.5 (23)
Non-Hispanic Black	20 293 (28 640)	7168 (55)	1104 (1963)	406 (58)	15.3 (4.1)	1.8 (14)	8.1 (4.4)	1.7 (27)
Non-Hispanic White	13 125 (19 819)	0 [Reference]	698 (1385)	0 [Reference]	13.5 (4.4)	0 [Reference]	6.4 (3.2)	0 [Reference]
Education								
>High school	15 284 (22 642)	0 [Reference]	784 (1489)	0 [Reference]	14.6 (4.4)	0 [Reference]	7.0 (3.3)	0 [Reference]
≤High school	18 127 (25 804)	2843 (19)	1022 (1833)	274 (37)	14.8 (4.8)	0.2 (2)	7.4 (3.8)	0.4 (5)
Place of birth								
US	15 268 (22 416)	0 [Reference]	839 (1587)	0 [Reference]	14.1 (4.5)	0 [Reference]	6.9 (3.6)	0 [Reference]
Non-US	22 332 (29824)	7064 (46)	1162 (2038)	323 (38)	16.7 (4.5)	2.6 (19)	8.2 (3.4)	1.3 (19)
Neighborhood HOLC grade^a^								
A or B	25 865 (31 447)	0 [Reference]	1400 (2423)	0 [Reference]	17.5 (3.4)	0 [Reference]	10.0 (3.6)	0 [Reference]
C or D	28 055 (32 986)	2190 (8)	1690 (2736)	290 (21)	18.5 (3.3)	1 (5)	11.1 (3.5)	1.1 (11)
Neighborhood-level income^b^								
High	13 422 (20 254)	0 [Reference]	666 (1355)	0 [Reference]	14.1 (4.3)	0 [Reference]	6.5 (3.0)	0 [Reference]
Middle	17 093 (23 732)	3671 (27)	937 (1658)	271 (41)	14.5 (4.8)	0.4 (3)	7.1 (3.8)	0.6 (10)
Low	20 762 (28 907)	7340 (55)	1164 (2044)	498 (75)	15.6 (4.8)	1.5 (11)	8.0 (3.9)	1.5 (23)
**2016**								
Race and ethnicity								
Hispanic or Latinx	17 919 (27 998)	5441 (44)	1043 (2033)	312 (43)	6.6 (2.7)	1.4 (25)	3.0 (1.5)	0.5 (23)
Non-Hispanic Asian or Pacific Islander	21 460 (31 175)	8982 (72)	1058 (2016)	327 (45)	6.5 (2.4)	1.3 (23)	3.3 (1.0)	0.8 (35)
Non-Hispanic Black	22 836 (32 844)	10 358 (83)	1313 (2363)	582 (79)	6.7 (2.4)	1.5 (28)	3.2 (1.2)	0.7 (30)
Non-Hispanic White	12 478 (22 870)	0 [Reference]	731 (1653)	0 [Reference]	5.2 (2.4)	0 [Reference]	2.5 (1.3)	0 [Reference]
Education								
>High school	15 766 (26 561)	0 [Reference]	874 (1855)	0 [Reference]	5.8 (2.5)	0 [Reference]	2.9 (1.3)	0 [Reference]
≤High school	18 230 (28 512)	2464 (16)	1090 (2091)	216 (25)	6.5 (2.7)	0.7 (11)	2.9 (1.5)	<0.1 (1)
Place of birth								
US	15 069 (25 277)	0 [Reference]	896 (1872)	0 [Reference]	5.9 (2.5)	0 [Reference]	2.7 (1.4)	0 [Reference]
Non-US	21 501 (31 997)	6432 (43)	1163 (2181)	267 (30)	6.8 (2.6)	0.9 (16)	3.3 (1.4)	0.6 (19)
Neighborhood HOLC grade^a^								
A or B	29 323 (37 483)	0 [Reference]	1553 (2635)	0 [Reference]	9.3 (2.2)	0 [Reference]	3.9 (1.4)	0 [Reference]
C or D	32 712 (43 513)	3389 (12)	2077 (3522)	524 (34)	10.0 (2.0)	0.7 (7)	4.3 (1.5)	0.4 (10)
Neighborhood-level income^b^								
High	13 531 (24 276)	0 [Reference]	708 (1658)	0 [Reference]	5.3 (2.1)	0 [Reference]	2.8 (1.1)	0 [Reference]
Middle	15 471 (25 178)	1940 (14)	898 (1788)	190 (27)	5.8 (2.5)	0.5 (8)	2.6 (1.4)	−0.2 (−6)
Low	21 633 (31 741)	8102 (60)	1308 (2341)	600 (85)	7.2 (2.7)	1.9 (35)	3.2 (1.6)	0.4 (13)

Abbreviations: HOLC, Home Owners’ Loan Corporation; NATA, National Air Toxics Assessment; no_2_, nitrogen dioxide; VMT, vehicle miles traveled.

^a^ Data are from the HOLC grades. A or B indicates the least risky (ie, not redlined) areas; C or D, most risky (ie, redlined) areas.

^b^ Data are from the US Census at the census tract level.

**Figure 2.**
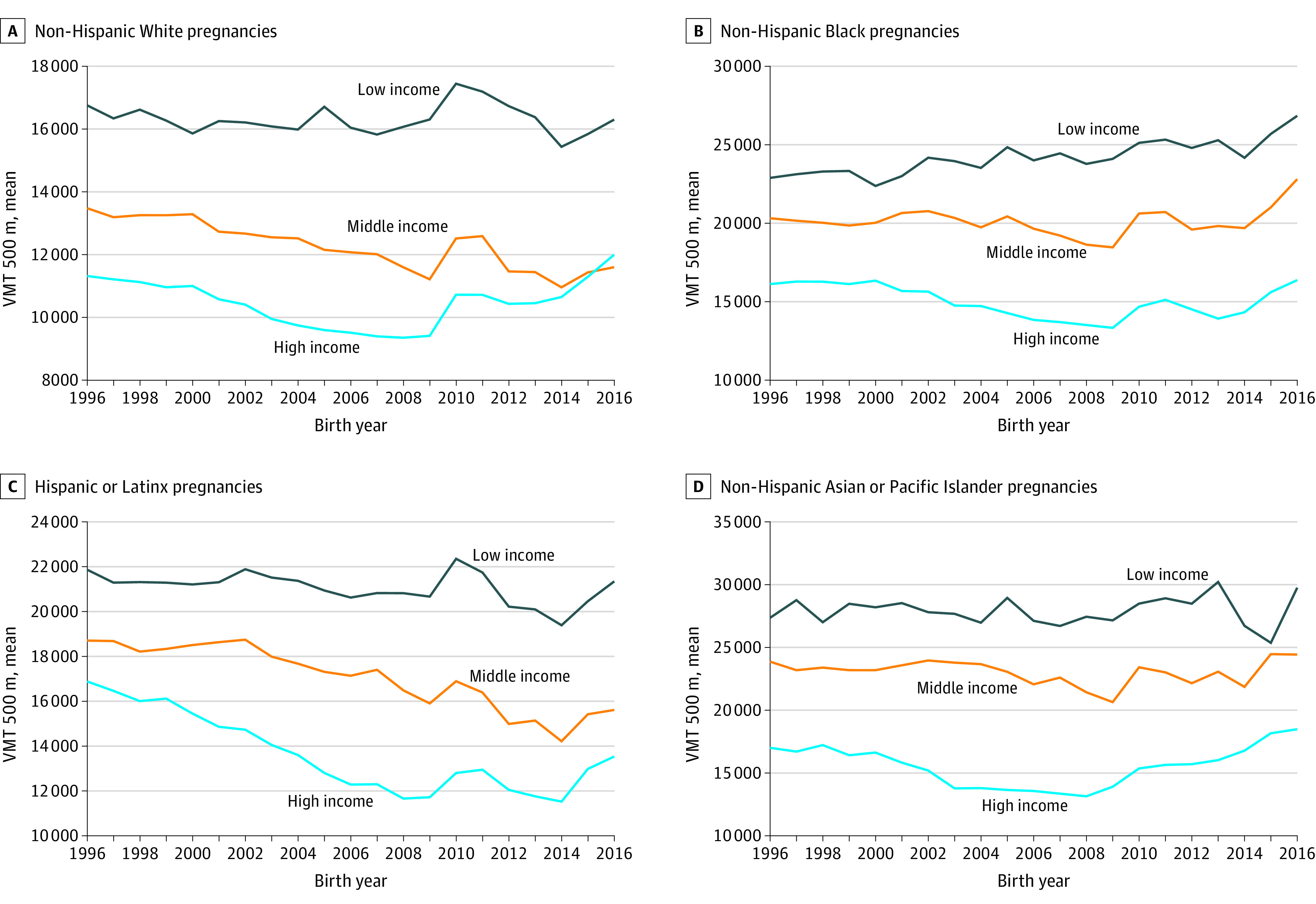
Summary of Vehicle Miles Traveled (VMT) Within 500 m Exposures by Individual Race and Ethnicity and Neighborhood Household Income Level, 1996-2016

When we stratified the population by race and ethnicity and neighborhood household income (Figure 2), we observed a consistent pattern of the lowest neighborhood household income category being exposed to the highest levels of VMT within 500 m for every racial and ethnic group, with little evidence of this disparity attenuated over time. Compared with pregnant non-Hispanic Black people, pregnant non-Hispanic White people were exposed to lower VMT within 500 m at every level of neighborhood household income. Results were similar, albeit attenuated, for other exposure metrics when stratified by neighborhood income categories (eFigures 4-6 in Supplement 1). Notably, pregnant non-Hispanic Black people in the highest neighborhood income tertile were exposed to similar levels of VMT as pregnant non-Hispanic White people in the lowest neighborhood income tertile, highlighting that neighborhood-level wealth did not account for differential exposure across race and ethnicity. This pattern of socioeconomic privilege and neighborhood household income was also present for other combinations of demographic characteristics and exposure metrics (eFigures 7-14 in Supplement 1).

We found distinct patterns in TRAP exposure disparities when we stratified the population by race and ethnicity and HOLC grade (eFigure 15 in Supplement 1). For all race ethnicity groups, except the Hispanic or Latinx group, we observed higher exposure to VMT within 500 m for the higher-graded neighborhoods (ie, C or D) compared with the lower-graded neighborhood (ie, A or B). The largest increase in this disparity was seen for pregnant non-Hispanic White people, while disparities between neighborhood disinvestment groups remained relatively stable for the other race and ethnicity groups. Results were similar, although attenuated, for other exposures (eFigures 16-18 in Supplement 1).

**Figure 3.**
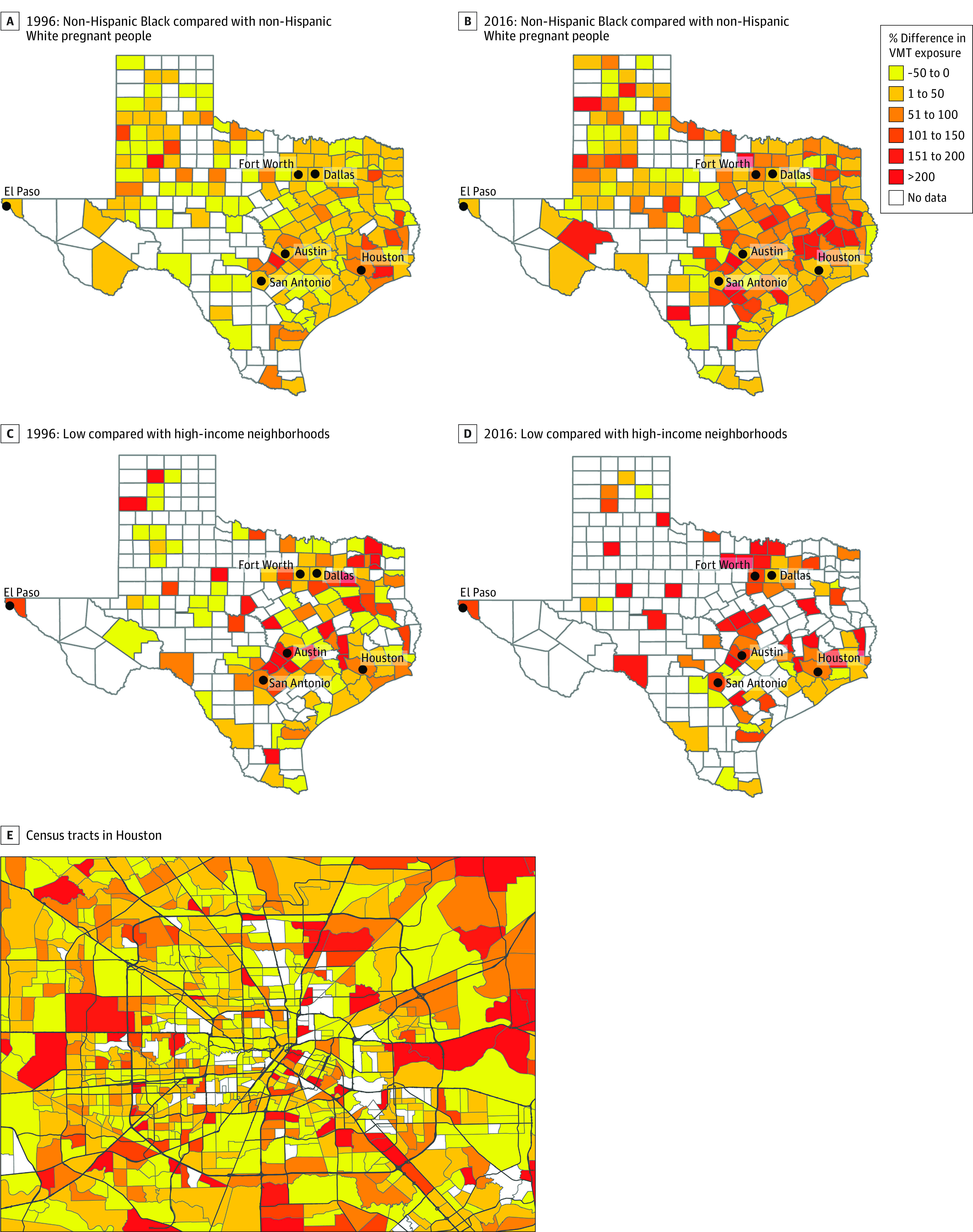
Percentage Differences in Vehicle Miles Traveled (VMT) Between Non-Hispanic White and Non-Hispanic Black Pregnant People and Low- and High-Income Census Tracts in Texas by County and Neighborhood in 1996 and 2016

We observed differences in TRAP exposure disparities and how these disparities changed over time by county and census tracts. At the county level, differences in VMT by race and ethnicity and neighborhood income were more concentrated in certain counties in 2016 compared with 1996 (Figure 3A-D). These disparities can also be seen at the census tract level or within-neighborhood level (Figure 3E), highlighting neighborhoods in Houston with the largest differences in VMT levels within 500m of Black compared with White pregnant people’s addresses in 2016.

## Discussion

This birth cohort study found that population-level exposures to TRAP substantially decreased between 1996 and 2016 among pregnant people in Texas. However, the magnitude of these improvements was consistently lower among persistently marginalized populations, such as Asian or Pacific Islander, Black, or Hispanic or Latinx individuals, and in lower-income neighborhoods. In addition, residential exposure to traffic has increased over time for pregnant non-Hispanic Black individuals, those with less than a high school diploma, those born outside the US, and those living in historically redlined or low-income neighborhoods, including disparities within neighborhoods. These results demonstrate the persistent legacy of structural sociodemographic segregation in Texas, highlighting the ongoing need for equitable implementation of environmental policy.

Large-scale transportation infrastructure projects often displace lower-income communities and disproportionately occur in areas with substantial populations of persistently marginalized people.^44^ In the United States, the siting of the interstate highway system dates back to the 1950s,^45^ around the time of systematic disinvestment in Black and Hispanic and Latinx communities via redlining in the 1930s.^46^ Although the Civil Rights Act of 1964 prohibited discrimination based on several socioeconomic and demographic characteristics soon afterward,^47^ infrastructure investments operate on a long-term time scale, as many road locations have not diverged from their original paths.^48^ We observed some of this legacy in our subanalysis of disparities by historical neighborhood disinvestment (ie, redlining), with evidence of persistent exposure inequities among the people who resided in the riskiest graded neighborhoods, per a policy from the 1930s.^46^ Surprisingly, the group that had the largest increase in exposures when restricted to the historical neighborhood disinvestment group was pregnant non-Hispanic White individuals, potentially unearthing the complexities of gentrification.^49^

Given that the locations of infrastructure rarely change, environmental justice concerns must be clearly considered before breaking ground on a new project, as the burden of pollution resulting from these projects can last generations. Concerns regarding the placement of transportation-related environmental hazards are becoming more mainstream,^50,51^ and some highways are being dismantled, rerouted, or reclaimed in favor of reducing the impact of TRAP on disproportionately burdened populations, including in Texas.^52,53^ As projects to dismantle transportation infrastructure are implemented, policy makers should also consider the real potential to induce a cycle of displacement and gentrification, which could in turn create worse environmental conditions for the very populations that the programs were aiming to protect.^54^ In this analysis, we found this phenomenon reflected in our data after stratification by census tract household income levels, where we saw consistent gradients of worse TRAP exposures in the lowest income neighborhoods, even when grouped by other individual characteristics.

Our results align with existing literature on the magnitudes of improvements in population exposures to air pollution, including TRAP, for people of lower socioeconomic positioning.^9,10,11^ For instance, national census data demonstrated that population-weighted mean concentrations of no_2_ were higher among Hispanic (11.0 ppb), non-Hispanic Asian or Pacific Islander (12.0 ppb), and non-Hispanic Black (9.7 ppb) communities compared with their non-Hispanic White counterparts (7.2 ppb) in 2010.^11^ Much of this disparity is reinforced by policy design, as seen in historically redlined areas.^14^ Previous work also shows that cancer risks from all air toxins are higher among non-Hispanic Black communities in Maryland,^55^ although this result is not directly comparable with our vehicle-specific metric. By using individual-level data from vital statistics, we confirmed persistence of the neighborhood-level results on TRAP exposure disparities for people of lower socioeconomic status and persistently marginalized groups.

Other metrics of TRAP (eg, all and truck-specific VMT) are not often assessed with respect to socioeconomic and racial disparities. While the large reductions in tailpipe emissions are a regulatory success (ie, no_2_, a commonly used indicator of tailpipe emissions, was markedly reduced between 1996 and 2016), disparities in the level of traffic around residential addresses increased. We also observed that truck-specific VMT disparities increased more than total VMT disparities. For example, truck VMT within 500 m was 58% higher for non-Hispanic Black pregnant people compared with non-Hispanic White people in 1996, and this difference increased to 79% in 2016. This increase was due primarily to more truck and total VMT on existing major roadways, rather than truck and vehicle traffic being routed to marginalized neighborhoods. Many detrimental exposures are associated with living in areas of higher traffic: increased traffic noise has been associated with a range of adverse health effects, including adverse birth outcomes^56^; there is increased air pollution from brake and tire wear, especially in stop-and-go traffic^57,58^; traffic congestion may be associated with adverse health outcomes^59,60^; crashes involving pedestrians increase with more vehicle traffic^61,62^; and high-traffic streets and neighborhoods are associated with less outdoor physical activity and a lower sense of belonging in community members.^63^ The totality of these traffic-related exposures has been associated with immediate risks to the pregnant person (eg, preeclampsia)^64,65^ and their infant (eg, preterm birth, term low birth weight),^60,66,67^ as well as long-term consequences for the dyad.^34,68^

A key challenge in environmental justice literature is selecting measures for evaluating inequity. An absolute measure (eg, difference) can be used for clinical and policy recommendations, while a relative difference (eg, percentage change) is trickier for efficient research translation.^69^ In our study, we found that the relative magnitude of the difference was stark—for instance, a 28% difference in no_2_ between non-Hispanic White and non-Hispanic Black pregnant people in 2016—but the absolute difference was not large (eg, a corresponding 1.5-ppb difference). Low levels of air pollution (ie, well below regulatory limits) have been associated with mortality,^70,71,72^ indicating that small differences in exposure have implications for population health.

Considering the socioeconomic characteristics related to the inequitable distribution of TRAP, future policy must be aimed at this important issue. Current policy strategies are likely to be ineffective for eliminating disparities in TRAP exposures, as shown by a recent analysis of the regulatory mechanisms for particulate matter of 2.5 microns or less (PM_2.5_) in the Clean Air Act.^20^ The transition to electric vehicles and further reductions in tailpipe emissions will result in lower TRAP exposures,^73,74,75^ but nontailpipe emissions (eg, brake and tire wear) will remain a concern.^76,77^ More importantly, however, is the double jeopardy of more vehicles in persistently marginalized neighborhoods in combination of preexisting environmental, social, and economic disadvantages.^78^ This goal will require long-term transportation planning that has environmental equity as a core driving principle.^20,79^ The Environmental Protection Agency’s Office of Environmental Justice and External Civil Rights has a goal of prioritizing equity, civil rights, and environmental justice principles into all practices, policies, and programs.^80^ Similar guiding principles are needed at local levels, where most land use policies are enacted. While characterizing disparities is an important first step, successful elimination of air pollution disparities, including TRAP, likely requires a radical reimagining of how environmental regulations are implemented.^20,21^

### Limitations

There are several limitations to consider with our results. First, TRAP metrics were measured at the annual level, precluding inferences in daily exposure patterns, and modeled exposure estimates include some degree of uncertainty. Second, some pollutants can travel beyond the 500 m range of VMT and aggregate downwind^81^; we were unable to evaluate exposures introduced this way, yielding a potential underestimate. Third, vital statistics data only provide address at delivery, a single time point during a highly mobile period in the life course,^82,83^ without any information on time activity patterns.^84^ Fourth, our data source was limited in what socioeconomic characteristics could be examined for the study period (1996-2016). Fifth, some exposure data sources (eg, HOLC) were not available for all pregnancies, given limited geographic scope. Sixth, we were unable to disentangle some of our sociodemographic characteristics in more depth, largely due to sample size considerations.^85^ For instance, we did not examine pregnancies among American Indian individuals, a group that is exposed to high concentrations of air pollution.^86^ Seventh, given that our analysis was predicated on reported characteristics related to residential locations and socioeconomic characteristics, we inherently cannot examine the pregnancies that were missing data, which may introduce selection bias.

## Conclusions

This birth cohort study found that although TRAP exposures during pregnancy decreased through the 20-year period of our study (1996-2016), the relative disparities between groups with higher and lower socioeconomic positioning largely increased over time. TRAP is an important environmental justice issue that affects pregnancy, and large disparities in traffic-related exposure levels remain, requiring renewed policy attention.

## Notes

Supplement 1. **eFigure 1.** Summary of no_2_ and VMT 500 m Exposures by Individual Birth Location and Neighborhood Income From 1996-2016 **eFigure 2.** Summary of Truck VMT 500 m and Vehicle Cancer Risk Exposures by Individual Race and Ethnicity and Educational Attainment From 1996-2016 **eFigure 3.** Summary of Truck VMT 500 m and Vehicle Cancer Risk Exposures by Individual Birth Location and Neighborhood Income From 1996-2016 **eFigure 4.** Summary of Truck VMT 500 m by Individual Race and Ethnicity and Neighborhood Income From 1996-2016 **eFigure 5.** Summary of no_2_ by Individual Race and Ethnicity and Neighborhood Income From 1996-2016 **eFigure 6.** Summary of Vehicle Cancer Risk by Individual Race and Ethnicity and Neighborhood Income From 1996-2016 **eFigure 7.** Summary of VMT 500 m by Individual Educational Attainment and Neighborhood Income From 1996-2016 **eFigure 8.** Summary of no_2_ by Individual Educational Attainment and Neighborhood Income From 1996-2016 **eFigure 9.** Summary of Truck VMT by Individual Educational Attainment and Neighborhood Income From 1996-2016 **eFigure 10.** Summary of Vehicle Cancer Risk by Individual Educational Attainment and Neighborhood Income From 1996-2016 **eFigure 11.** Summary of VMT 500 m by Individual Birthplace and Neighborhood Income From 1996-2016 **eFigure 12.** Summary of no_2_ by Individual Birthplace and Neighborhood Income From 1996-2016 **eFigure 13.** Summary of Truck VMT 500 m by Individual Birthplace and Neighborhood Income From 1996-2016 **eFigure 14.** Summary of Vehicle Cancer Risk by Individual Birthplace and Neighborhood Income From 1996-2016 **eFigure 15.** Summary of VMT 500 m Exposures by Individual Race and Ethnicity and Historical Neighborhood Disinvestment From 1996-2016 **eFigure 16.** Summary of no_2_ by Individual Race and Ethnicity and Historical Neighborhood Disinvestment From 1996-2016 **eFigure 17.** Summary of Truck VMT 500 m by Individual Race and Ethnicity and Historical Neighborhood Disinvestment From 1996-2016 **eFigure 18.** Summary of Vehicle Cancer Risk by Individual Race and Ethnicity and Historical Neighborhood Disinvestment From 1996-2016

Supplement 2.
Data Sharing Statement
