# Supplementary material for: Changes in Socioeconomic Disparities for Traffic-Related Air Pollution Exposure During Pregnancy Over a 20-Year Period in Texas
Source: JAMA Netw Open. 2023 Aug 11;6(8):e2328012. doi: 10.1001/jamanetworkopen.2023.28012 (PMC10422188; doi:10.1001/jamanetworkopen.2023.28012)
Supplement: Supplement 1. — eFigure 1. Summary of no2 and VMT 500 m Exposures by Individual Birth Location and Neighborhood Income From 1996-2016 eFigure 2. Summary of Truck VMT 500 m and Vehicle Cancer Risk Exposures by Individual Race and Ethnicity and Educational Attainment From 1996-2016 eFigure 3. Summary of Truck VMT 500 m and Vehicle Cancer Risk Exposures by Individual Birth Location and Neighborhood Income From 1996-2016 eFigure 4. Summary of Truck VMT 500 m by Individual Race and Ethnicity and Neighborhood Income From 1996-2016 eFigure 5. Summary of no2 by Individual Race and Ethnicity and Neighborhood Income From 1996-2016 eFigure 6. Summary of Vehicle Cancer Risk by Individual Race and Ethnicity and Neighborhood Income From 1996-2016 eFigure 7. Summary of VMT 500 m by Individual Educational Attainment and Neighborhood Income From 1996-2016 eFigure 8. Summary of no2 by Individual Educational Attainment and Neighborhood Income From 1996-2016 eFigure 9. Summary of Truck VMT by Individual Educational Attainment and Neighborhood Income From 1996-2016 eFigure 10. Summary of Vehicle Cancer Risk by Individual Educational Attainment and Neighborhood Income From 1996-2016 eFigure 11. Summary of VMT 500 m by Individual Birthplace and Neighborhood Income From 1996-2016 eFigure 12. Summary of no2 by Individual Birthplace and Neighborhood Income From 1996-2016 eFigure 13. Summary of Truck VMT 500 m by Individual Birthplace and Neighborhood Income From 1996-2016 eFigure 14. Summary of Vehicle Cancer Risk by Individual Birthplace and Neighborhood Income From 1996-2016 eFigure 15. Summary of VMT 500 m Exposures by Individual Race and Ethnicity and Historical Neighborhood Disinvestment From 1996-2016 eFigure 16. Summary of no2 by Individual Race and Ethnicity and Historical Neighborhood Disinvestment From 1996-2016 eFigure 17. Summary of Truck VMT 500 m by Individual Race and Ethnicity and Historical Neighborhood Disinvestment From 1996-2016 eFigure 18. Summary of Vehicle Cancer Risk by Individual Ra [file jamanetwopen-e2328012-s001.pdf]

# Supplemental Online Content

Willis MD, Hill EL, Ncube CN, et al. Changes in socioeconomic disparities for traffic-related air pollution exposure during pregnancy over a 20-year period in Texas. *JAMA Netw Open*. 2023;6(8):e2328012. doi:10.1001/jamanetworkopen.2023.28012

**eFigure 1.** Summary of $NO_2$ and VMT 500 m Exposures by Individual Birth Location and Neighborhood Income From 1996-2016
**eFigure 2.** Summary of Truck VMT 500 m and Vehicle Cancer Risk Exposures by Individual Race and Ethnicity and Educational Attainment From 1996-2016
**eFigure 3.** Summary of Truck VMT 500 m and Vehicle Cancer Risk Exposures by Individual Birth Location and Neighborhood Income From 1996-2016
**eFigure 4.** Summary of Truck VMT 500 m by Individual Race and Ethnicity and Neighborhood Income From 1996-2016
**eFigure 5.** Summary of $NO_2$ by Individual Race and Ethnicity and Neighborhood Income From 1996-2016
**eFigure 6.** Summary of Vehicle Cancer Risk by Individual Race and Ethnicity and Neighborhood Income From 1996-2016
**eFigure 7.** Summary of VMT 500 m by Individual Educational Attainment and Neighborhood Income From 1996-2016
**eFigure 8.** Summary of $NO_2$ by Individual Educational Attainment and Neighborhood Income From 1996-2016
**eFigure 9.** Summary of Truck VMT by Individual Educational Attainment and Neighborhood Income From 1996-2016
**eFigure 10.** Summary of Vehicle Cancer Risk by Individual Educational Attainment and Neighborhood Income From 1996-2016
**eFigure 11.** Summary of VMT 500 m by Individual Birthplace and Neighborhood Income From 1996-2016
**eFigure 12.** Summary of $NO_2$ by Individual Birthplace and Neighborhood Income From 1996-2016
**eFigure 13.** Summary of Truck VMT 500 m by Individual Birthplace and Neighborhood Income From 1996-2016
**eFigure 14.** Summary of Vehicle Cancer Risk by Individual Birthplace and Neighborhood Income From 1996-2016
**eFigure 15.** Summary of VMT 500 m Exposures by Individual Race and Ethnicity and Historical Neighborhood Disinvestment From 1996-2016
**eFigure 16.** Summary of $NO_2$ by Individual Race and Ethnicity and Historical Neighborhood Disinvestment From 1996-2016
**eFigure 17.** Summary of Truck VMT 500 m by Individual Race and Ethnicity and Historical Neighborhood Disinvestment From 1996-2016
**eFigure 18.** Summary of Vehicle Cancer Risk by Individual Race and Ethnicity and Historical Neighborhood Disinvestment From 1996-2016

This supplemental material has been provided by the authors to give readers additional information about their work.

© 2023 Willis MD et al. *JAMA Network Open.*

**Supplemental Figure 1:** Summary of $NO_2$ and VMT 500 m exposures by individual birth location and neighborhood income from 1996-2016. VMT: vehicle miles traveled. $NO_2$: nitrogen dioxide. PPB: parts per billion.

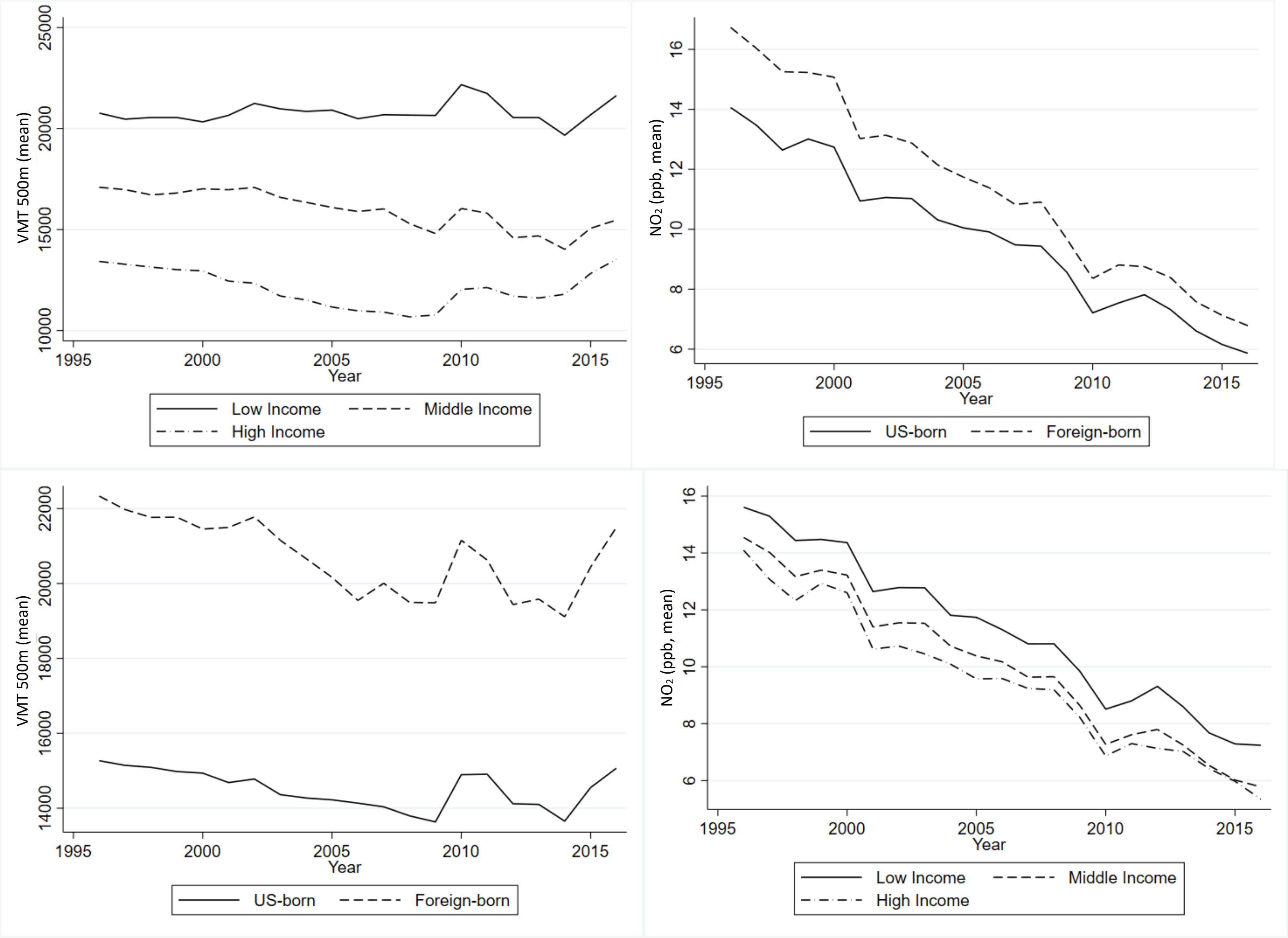


© 2023 Willis MD et al. *JAMA Network Open.*

**Supplemental Figure 2**: Summary of truck VMT 500 m and vehicle cancer risk exposures by individual race/ethnicity and educational attainment from 1996-2016. VMT: vehicle miles traveled.

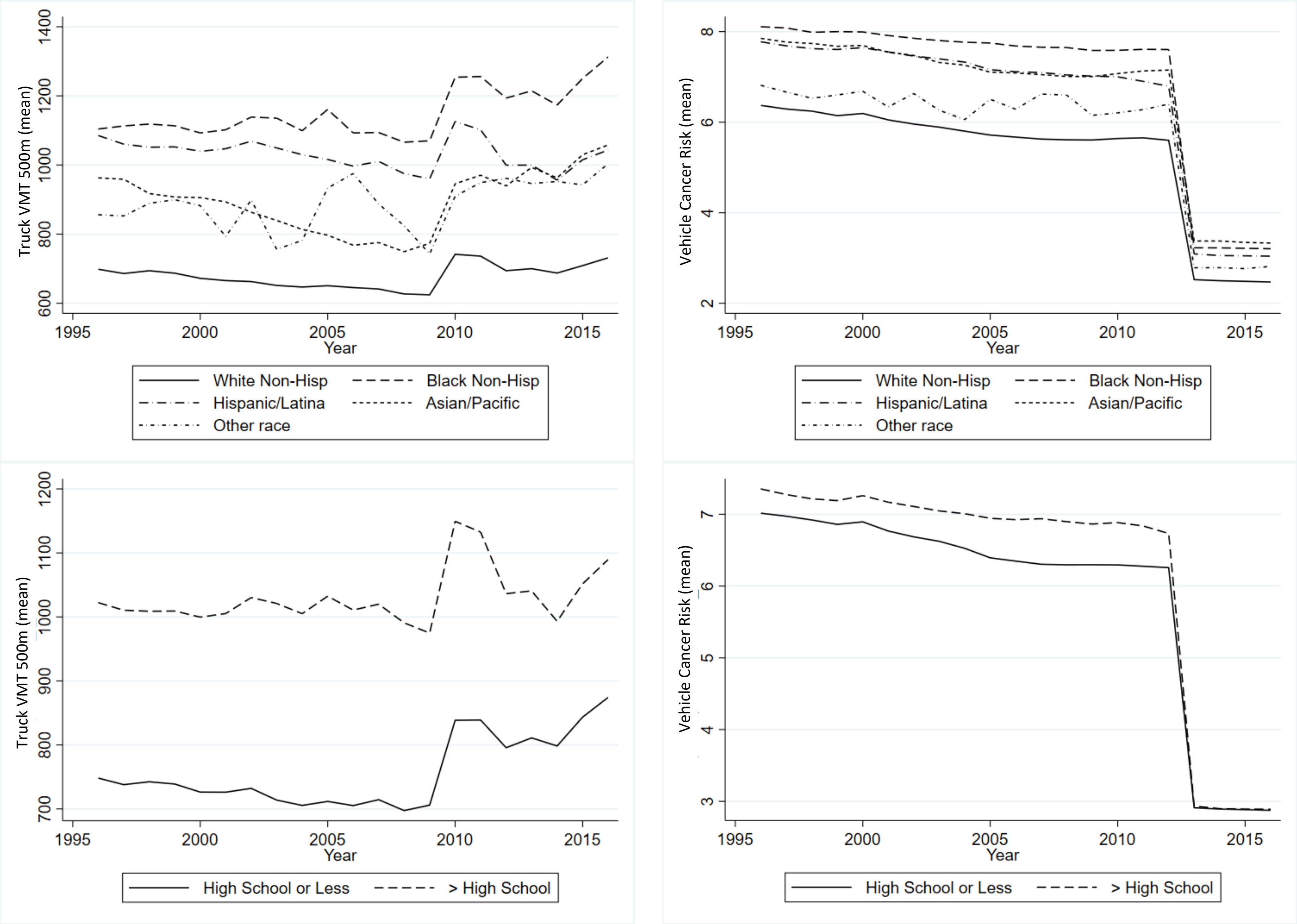


© 2023 Willis MD et al. *JAMA Network Open.*

**Supplemental Figure 3**: Summary of truck VMT 500 m and vehicle cancer risk exposures by individual birth location and neighborhood income from 1996-2016. VMT: vehicle miles traveled.

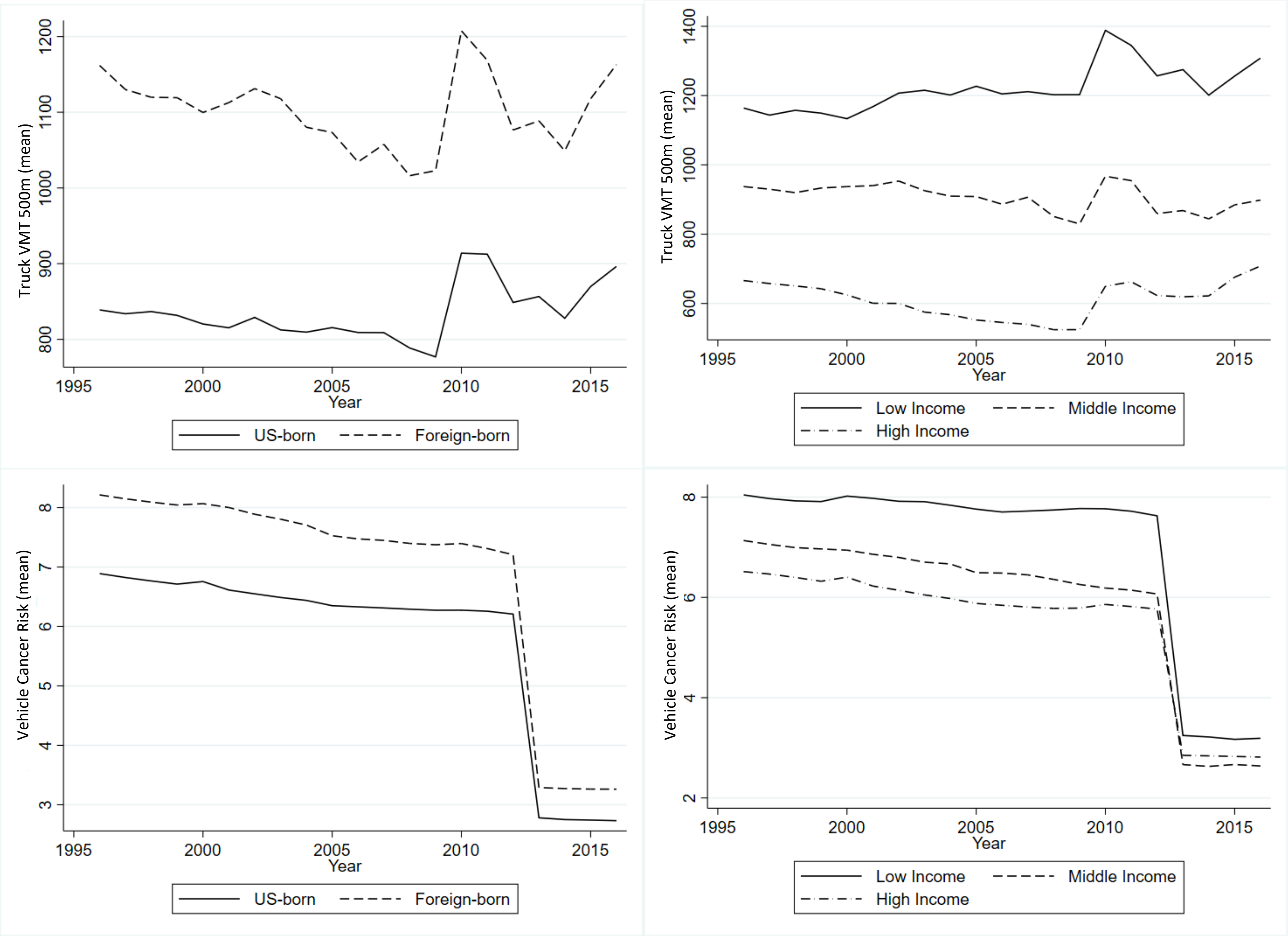


© 2023 Willis MD et al. *JAMA Network Open.*

**Supplemental Figure 4**: Summary of truck VMT 500 m by individual race/ethnicity and neighborhood income from 1996-2016. VMT: vehicle miles traveled.

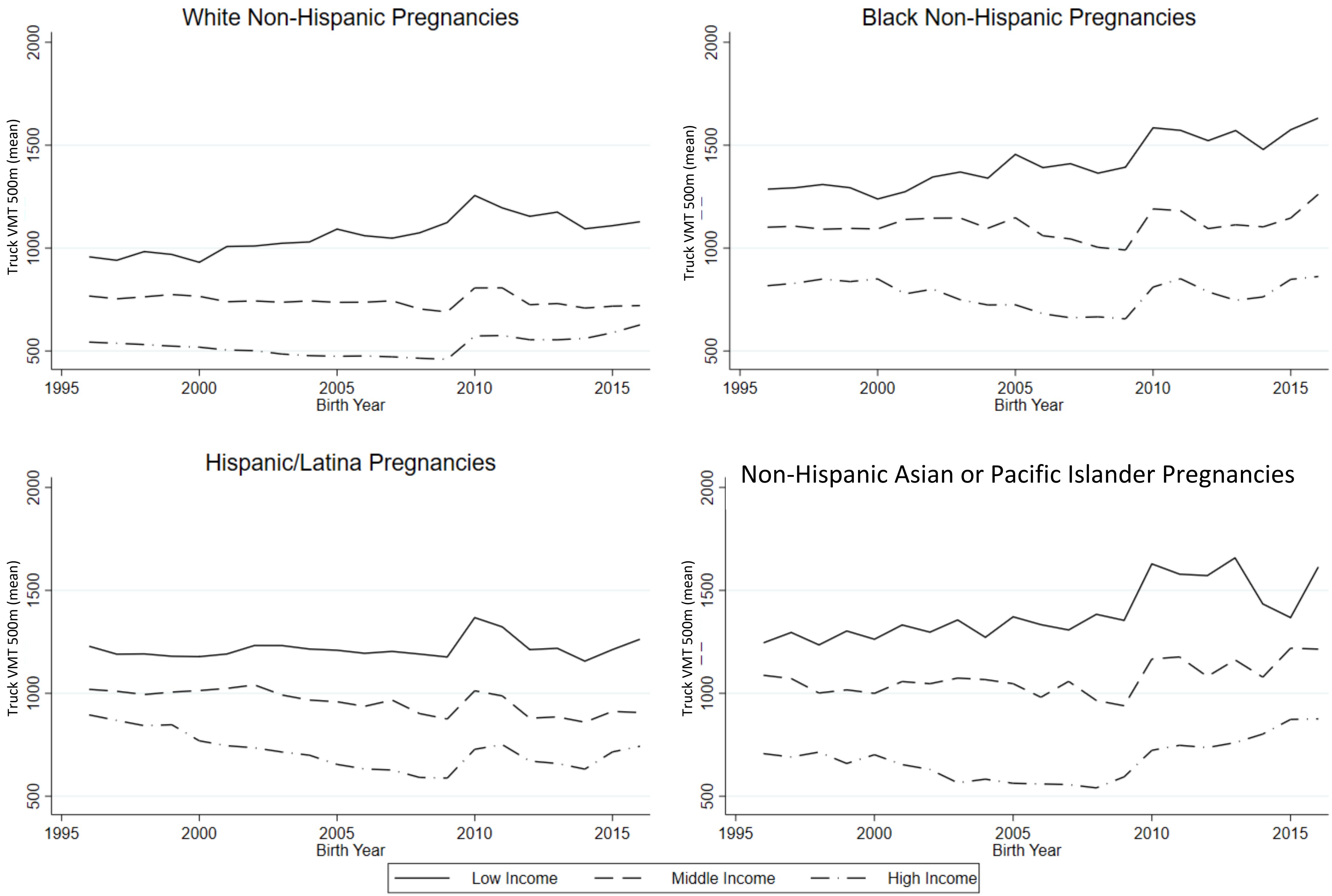


© 2023 Willis MD et al. *JAMA Network Open.*

**Supplemental Figure 5**: Summary of $NO_2$ by individual race/ethnicity and neighborhood income from 1996-2016. $NO_2$: nitrogen dioxide. PPB: parts per billion.

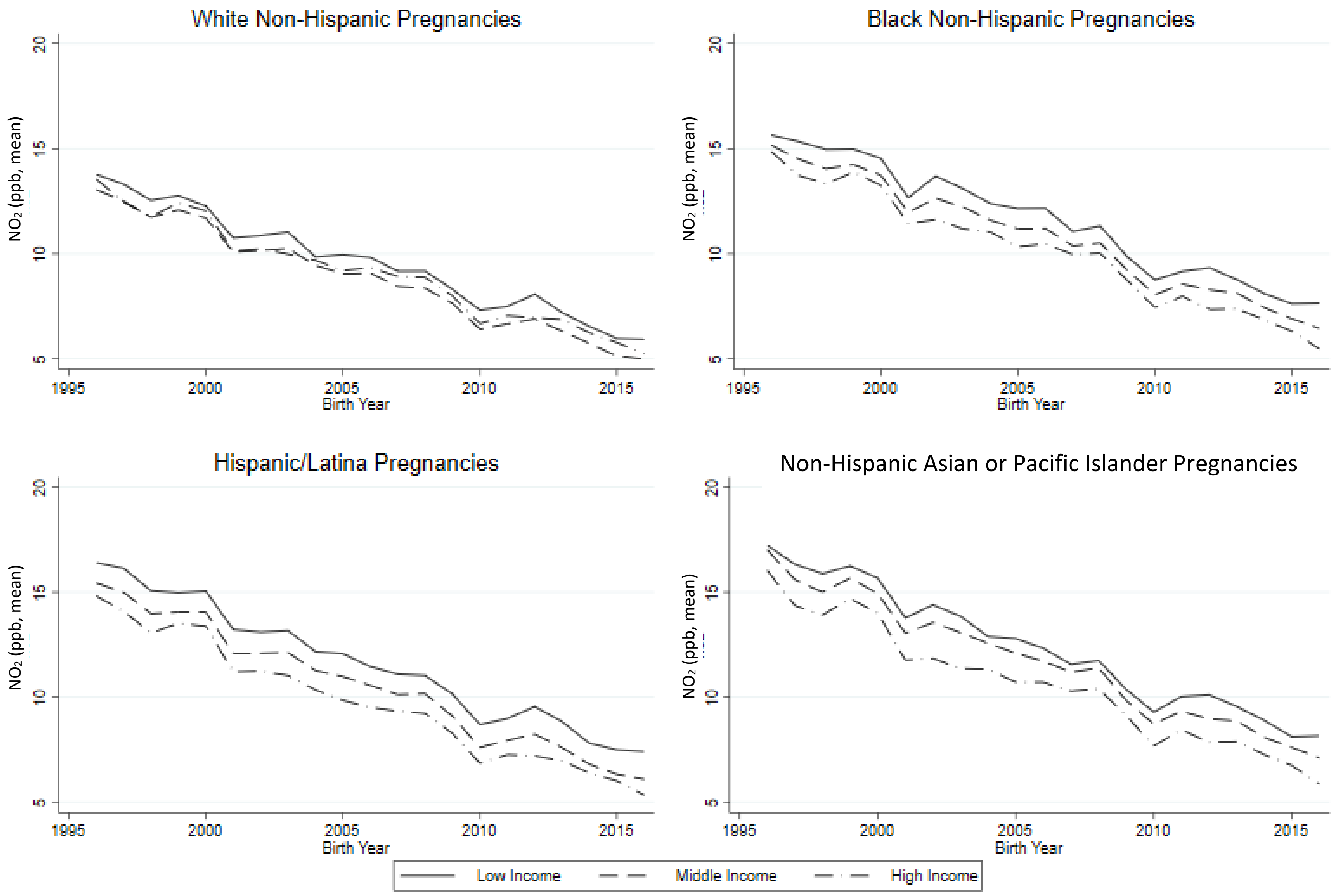


© 2023 Willis MD et al. *JAMA Network Open.*

**Supplemental Figure 6**: Summary of vehicle cancer risk by individual race/ethnicity and neighborhood income from 1996-2016

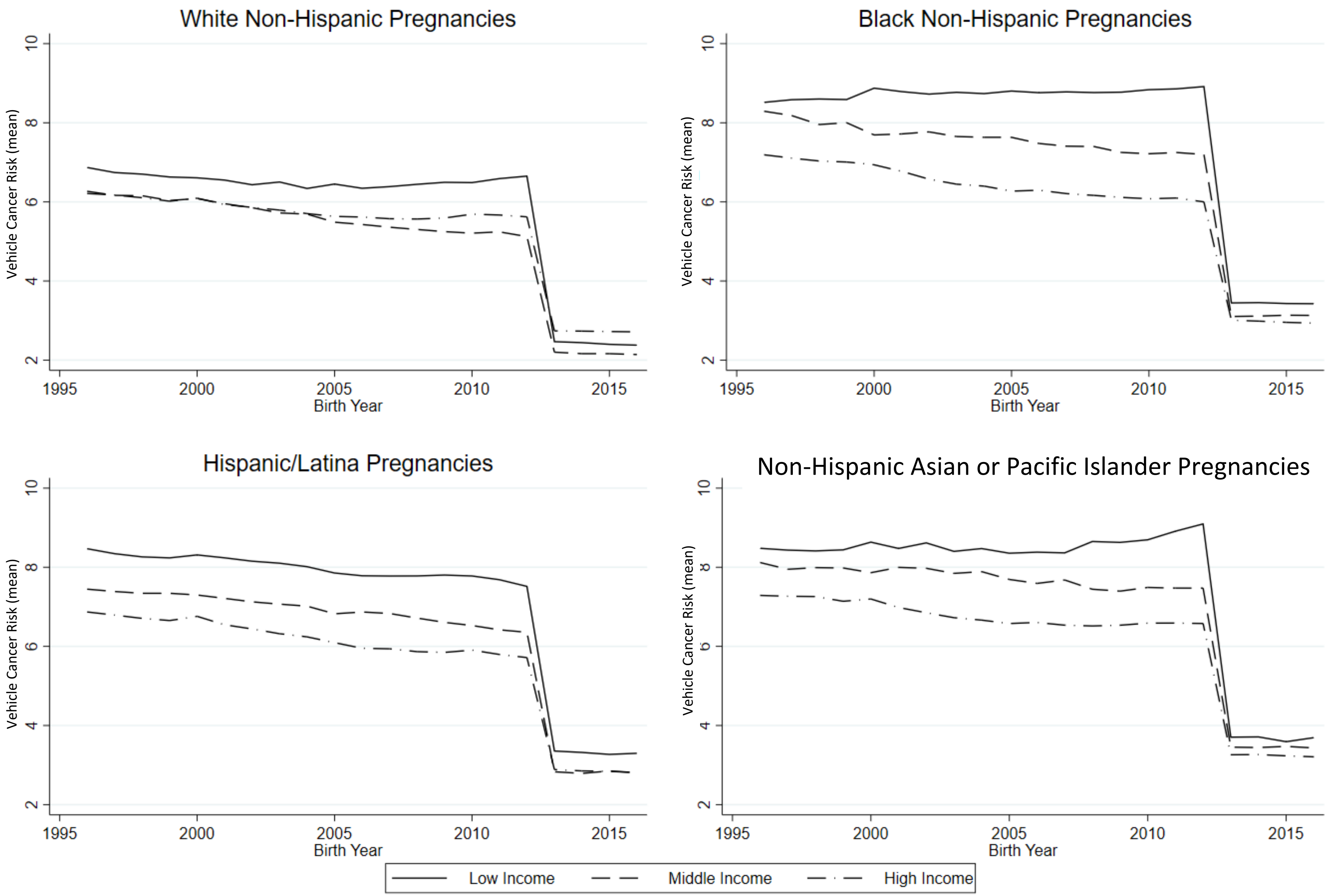


© 2023 Willis MD et al. *JAMA Network Open.*

**Supplemental Figure 7**: Summary of VMT 500 m by individual educational attainment and neighborhood income from 1996-2016. VMT: vehicle miles traveled.

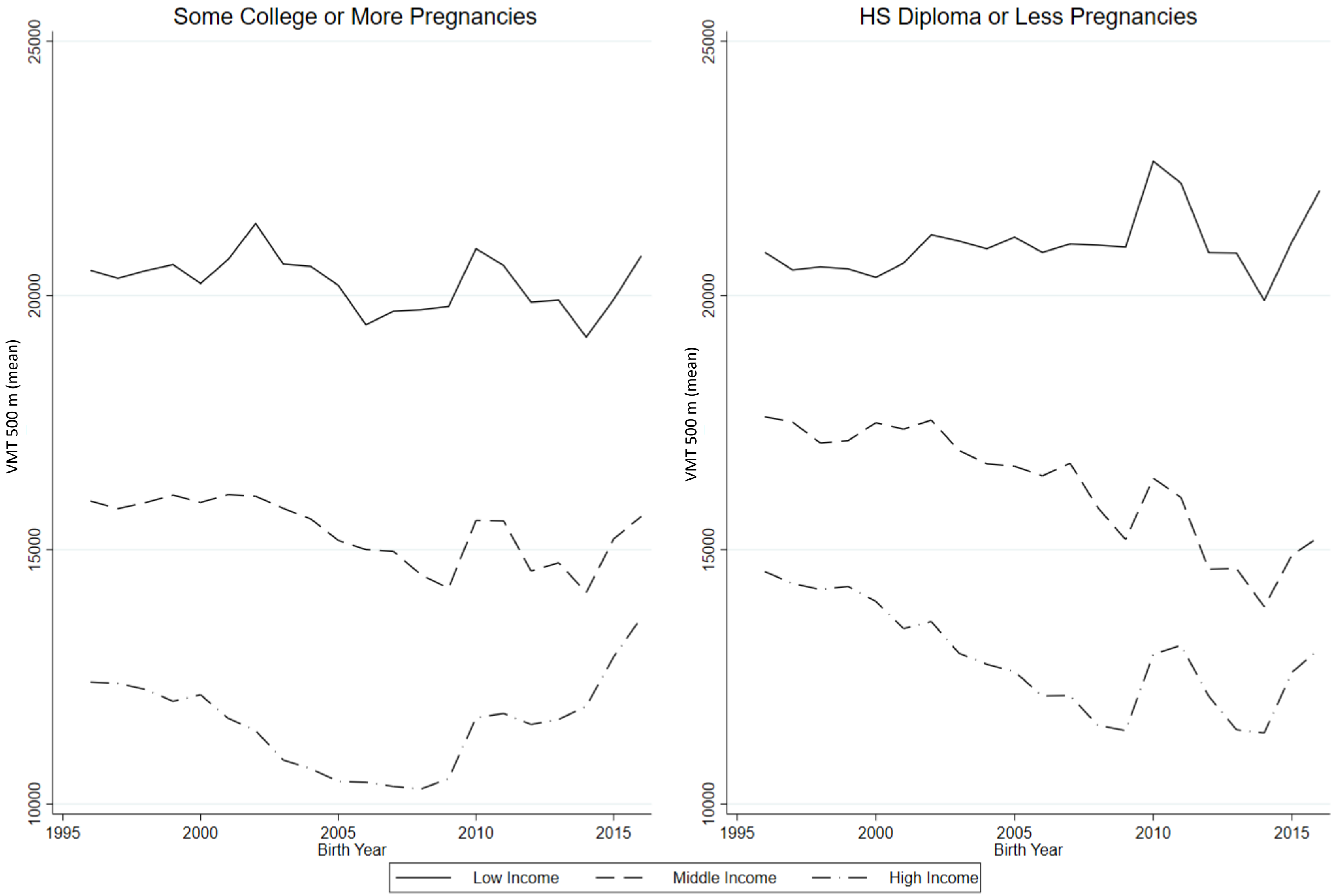


© 2023 Willis MD et al. *JAMA Network Open.*

**Supplemental Figure 8**: Summary of $NO_2$ by individual educational attainment and neighborhood income from 1996-2016. $NO_2$: nitrogen dioxide. PPB: parts per billion.

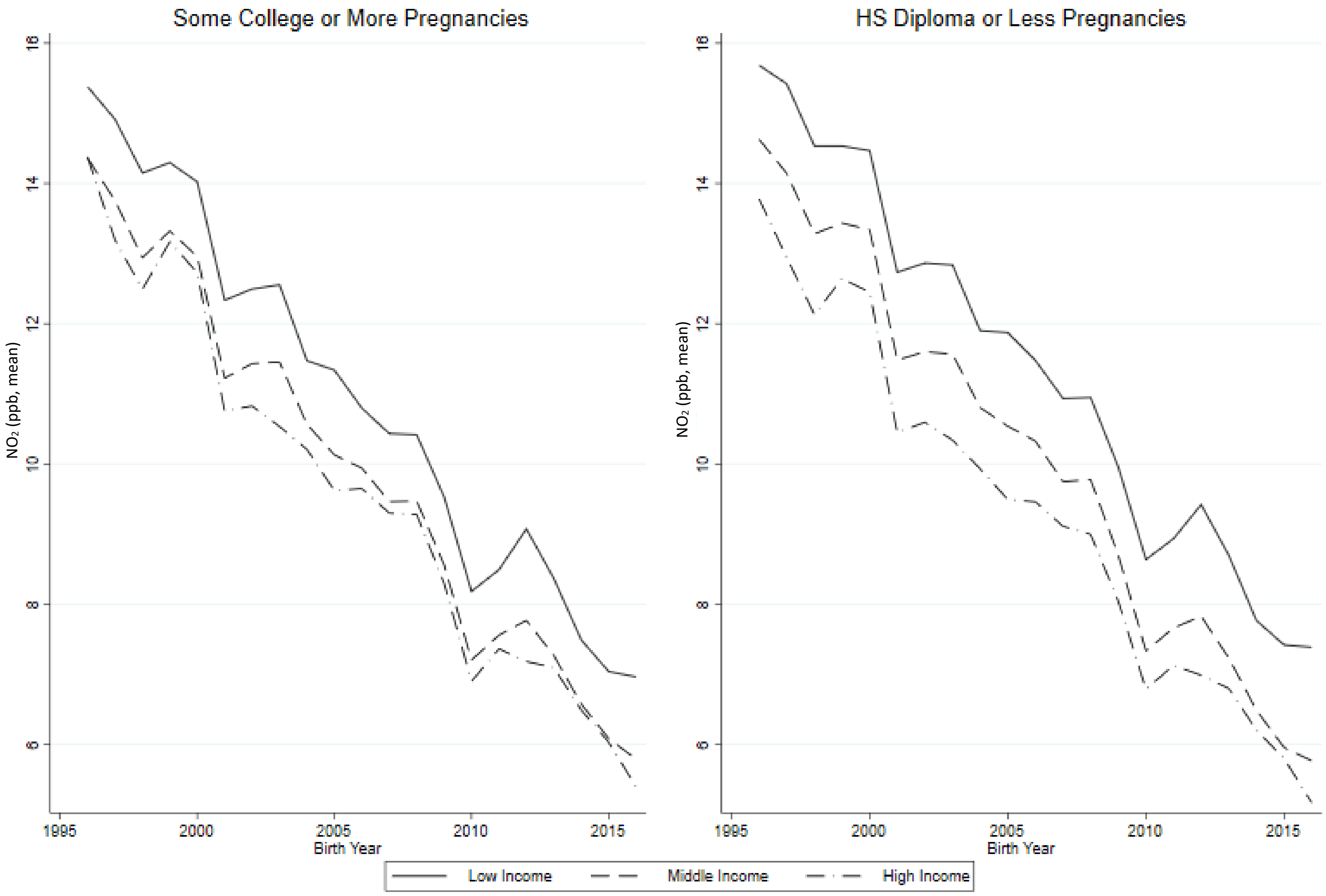


© 2023 Willis MD et al. *JAMA Network Open.*

**Supplemental Figure 9**: Summary of truck VMT by individual educational attainment and neighborhood income from 1996-2016. VMT: vehicle miles traveled.

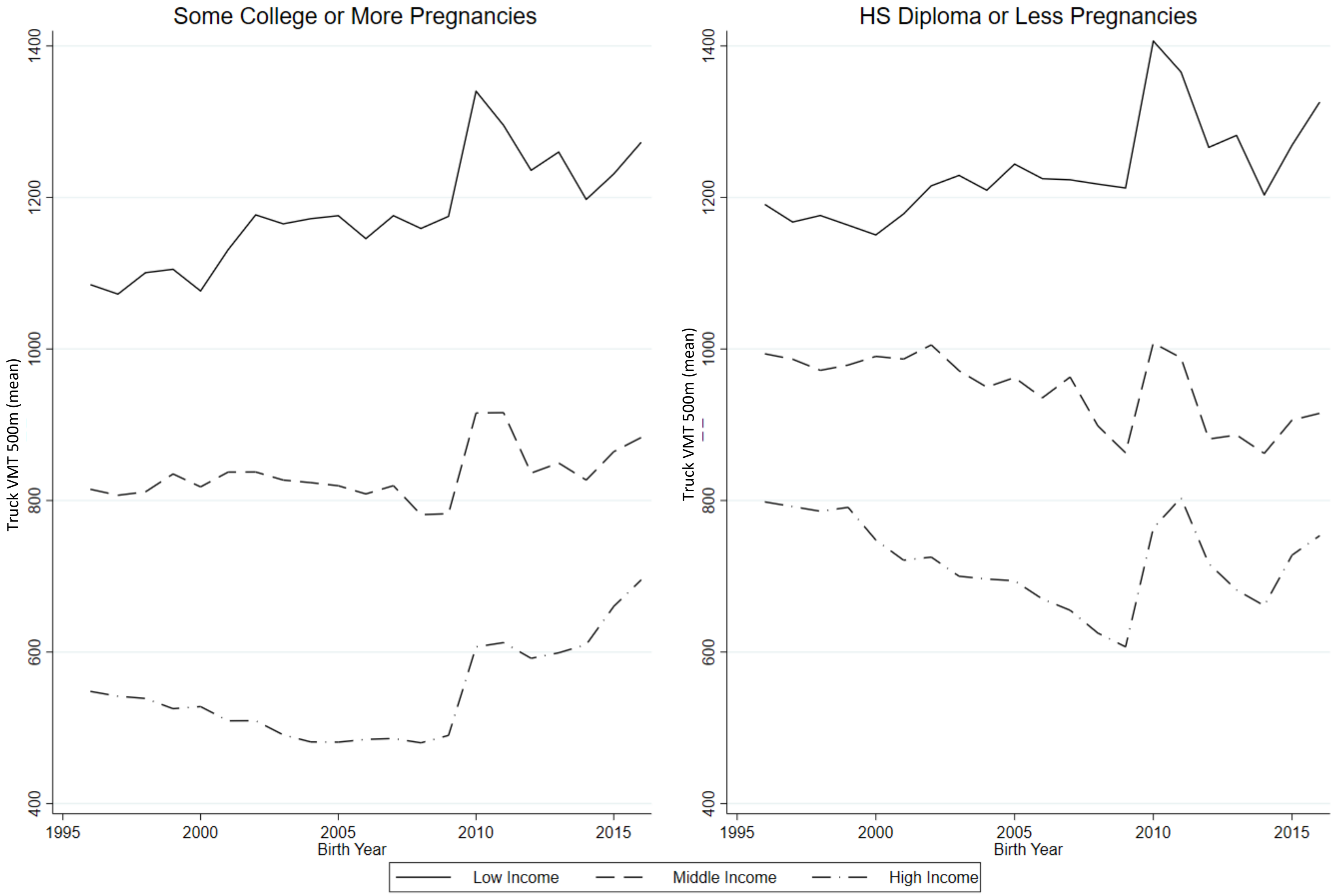


© 2023 Willis MD et al. *JAMA Network Open.*

**Supplemental Figure 10**: Summary of vehicle cancer risk by individual educational attainment and neighborhood income from 1996-2016

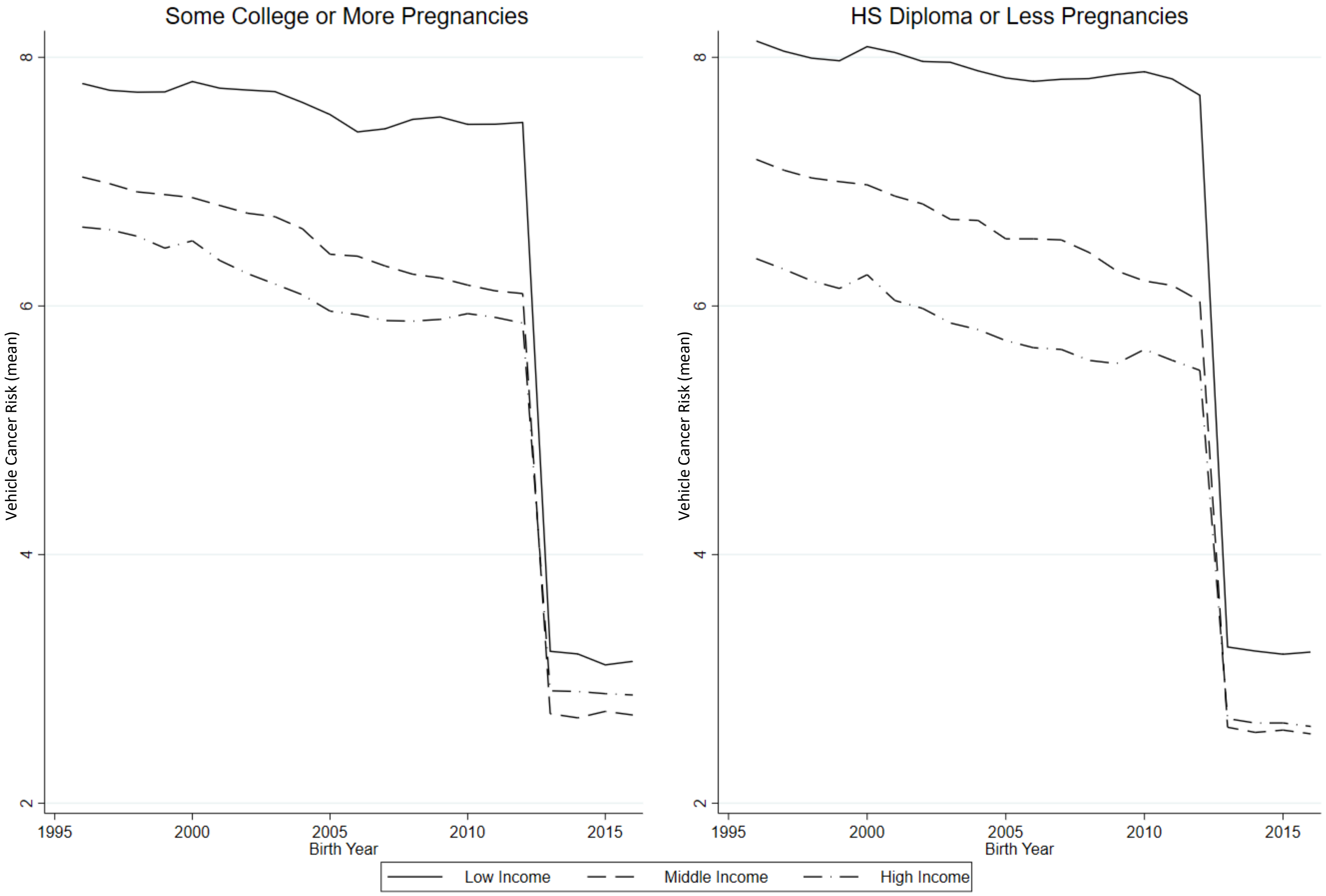


© 2023 Willis MD et al. *JAMA Network Open.*

**Supplemental Figure 11**: Summary of VMT 500 m by individual birthplace and neighborhood income from 1996-2016. VMT: vehicle miles traveled.

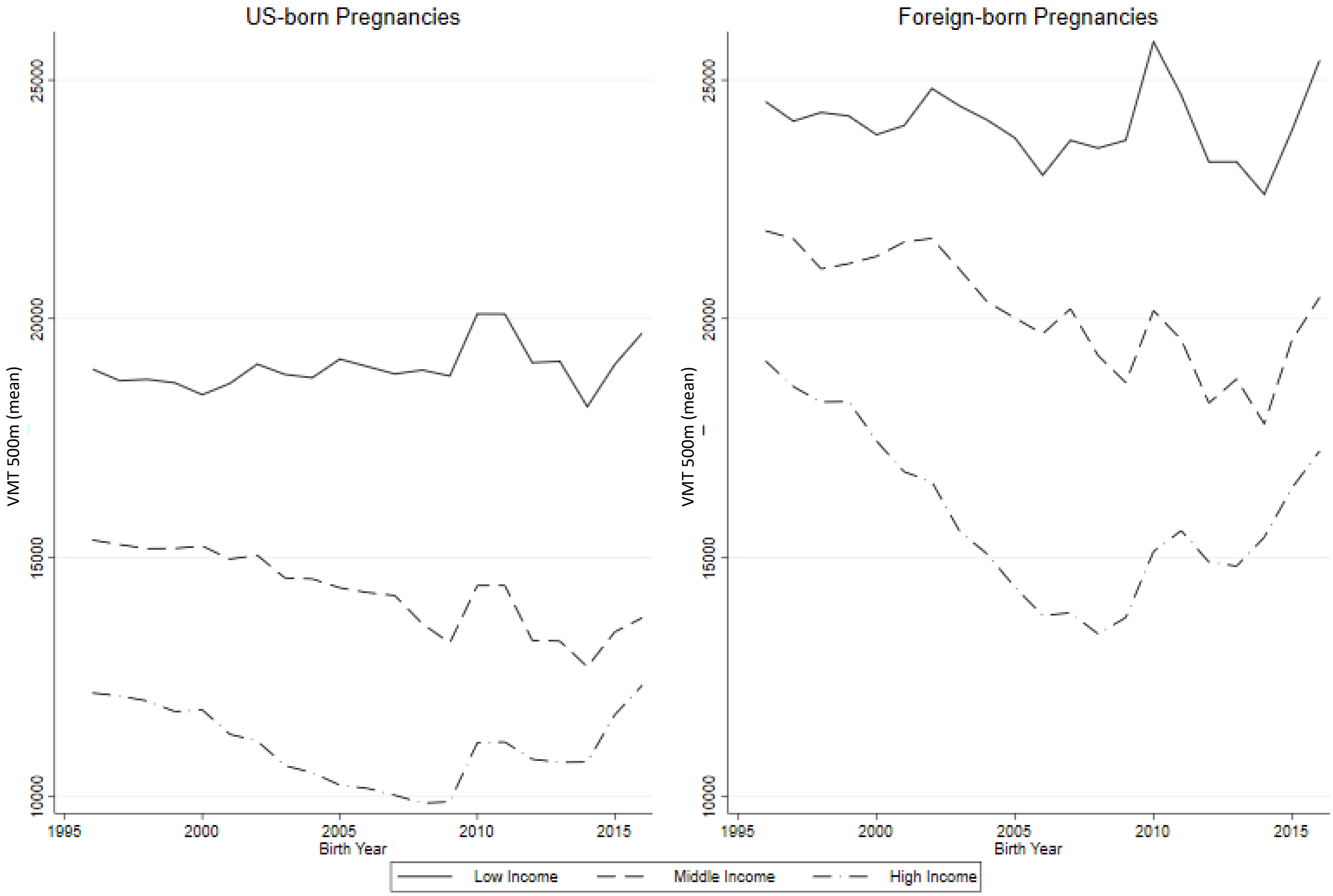


© 2023 Willis MD et al. *JAMA Network Open.*

**Supplemental Figure 12**: Summary of $NO_2$ by individual birthplace and neighborhood income from 1996-2016. $NO_2$: nitrogen dioxide. PPB: parts per billion.

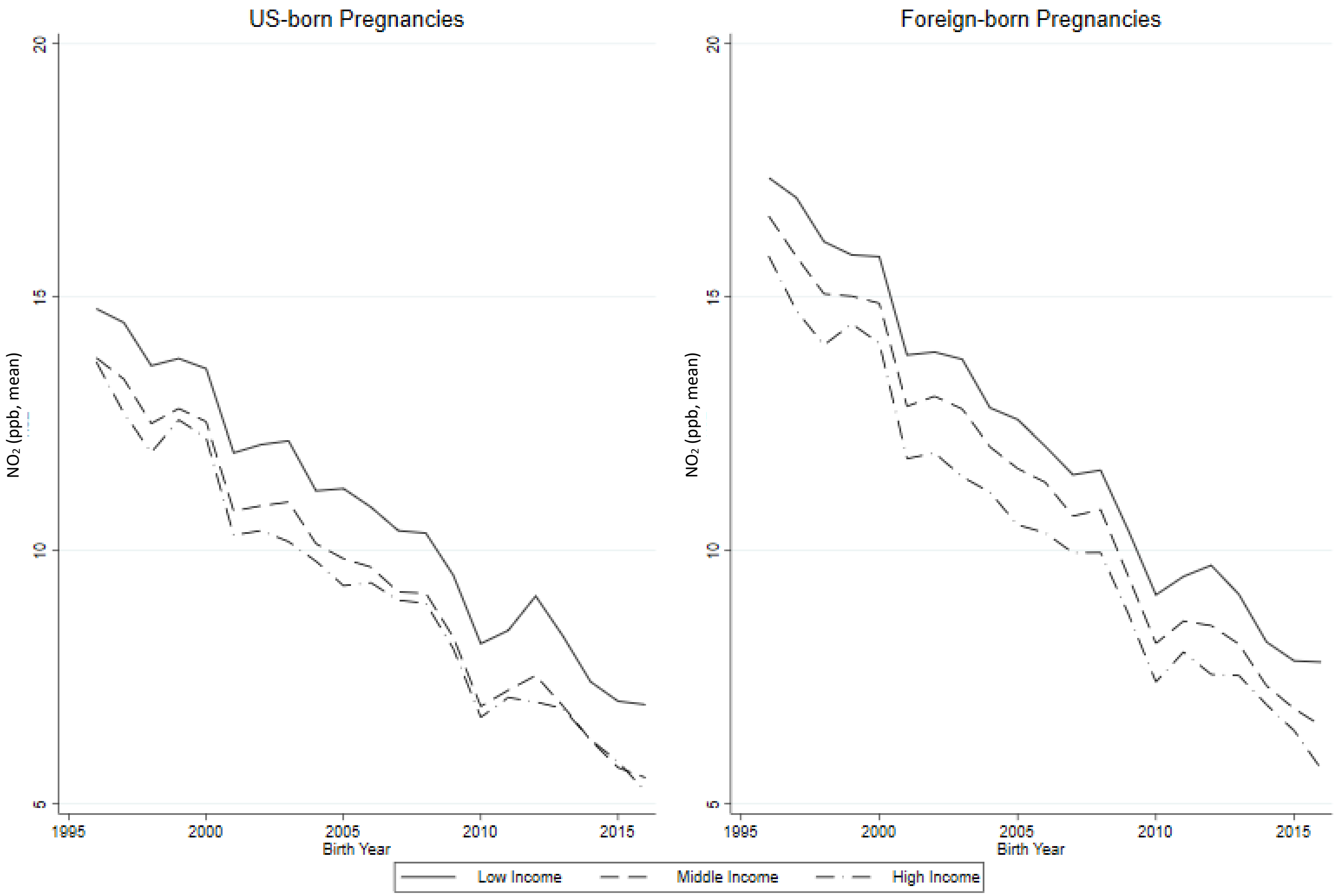


© 2023 Willis MD et al. *JAMA Network Open.*

**Supplemental Figure 13**: Summary of truck VMT 500 m by individual birthplace and neighborhood income from 1996-2016. VMT: vehicle miles traveled.

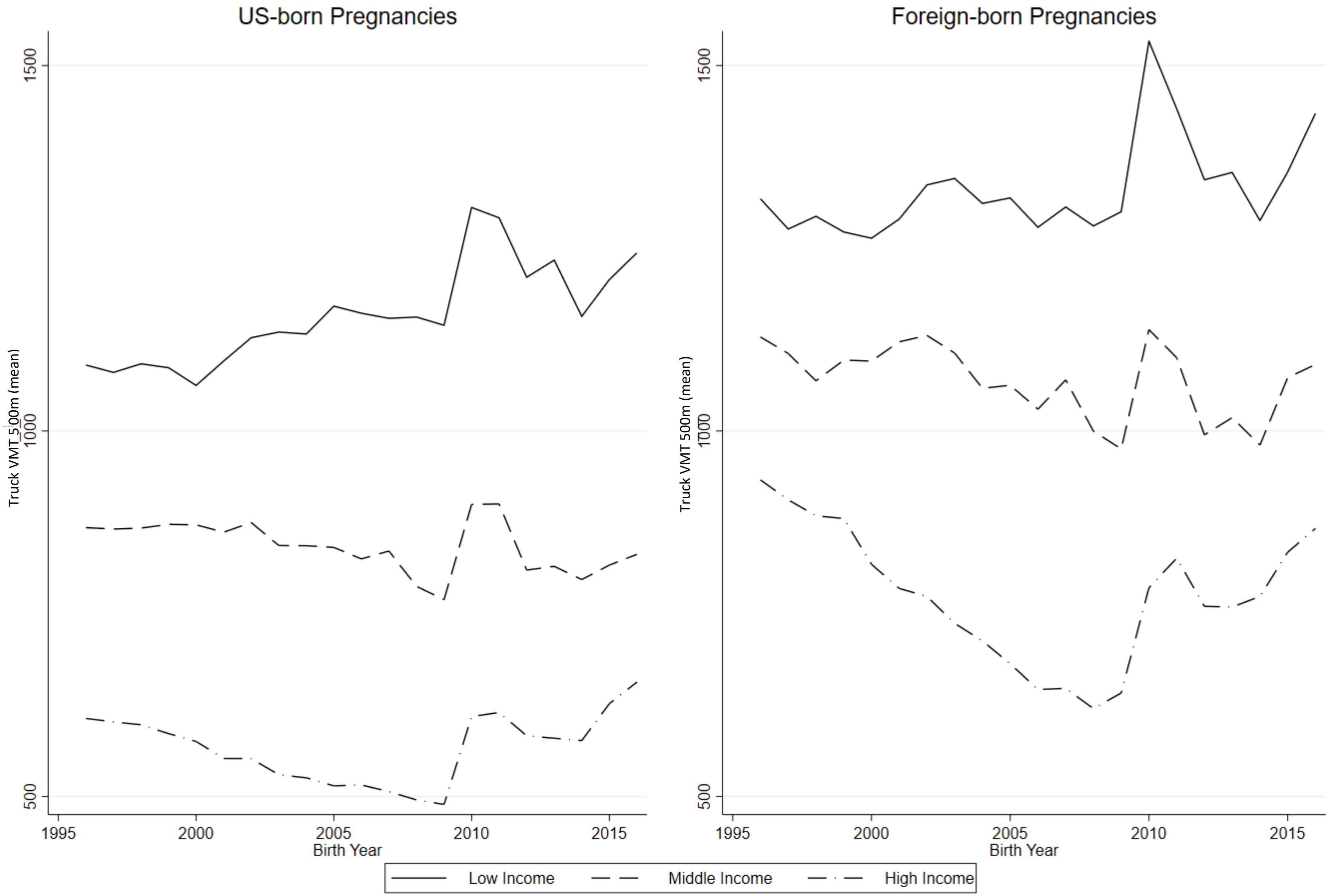


© 2023 Willis MD et al. *JAMA Network Open.*

**Supplemental Figure 14**: Summary of vehicle cancer risk by individual birthplace and neighborhood income from 1996-2016

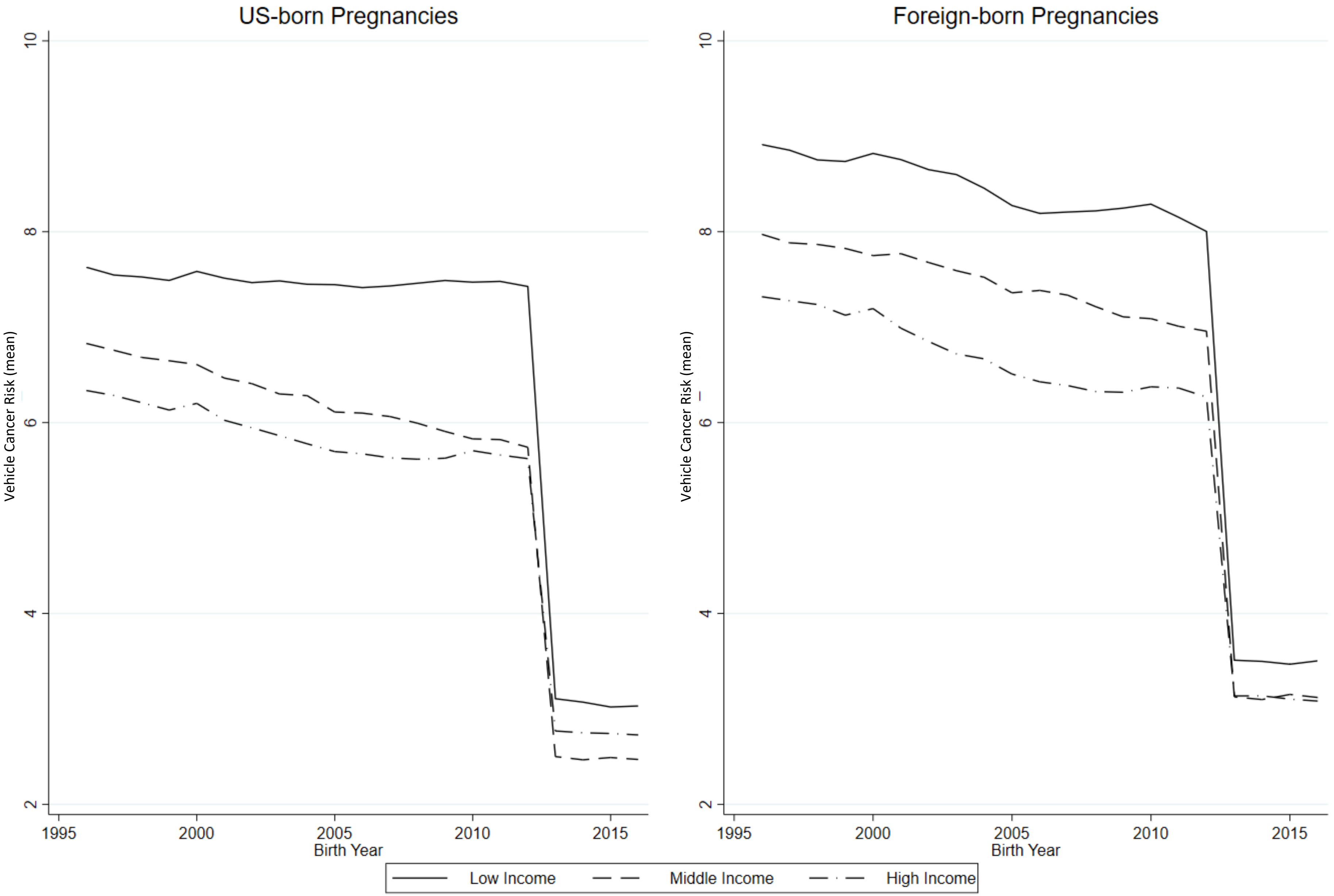


© 2023 Willis MD et al. *JAMA Network Open.*

**Supplemental Figure 15**: Summary of VMT 500 m exposures by individual race/ethnicity and historical neighborhood disinvestment from 1996-2016. A or B: the “least risky” (i.e., not redlined) areas; C or D: the “most risky” (i.e., redlined) areas

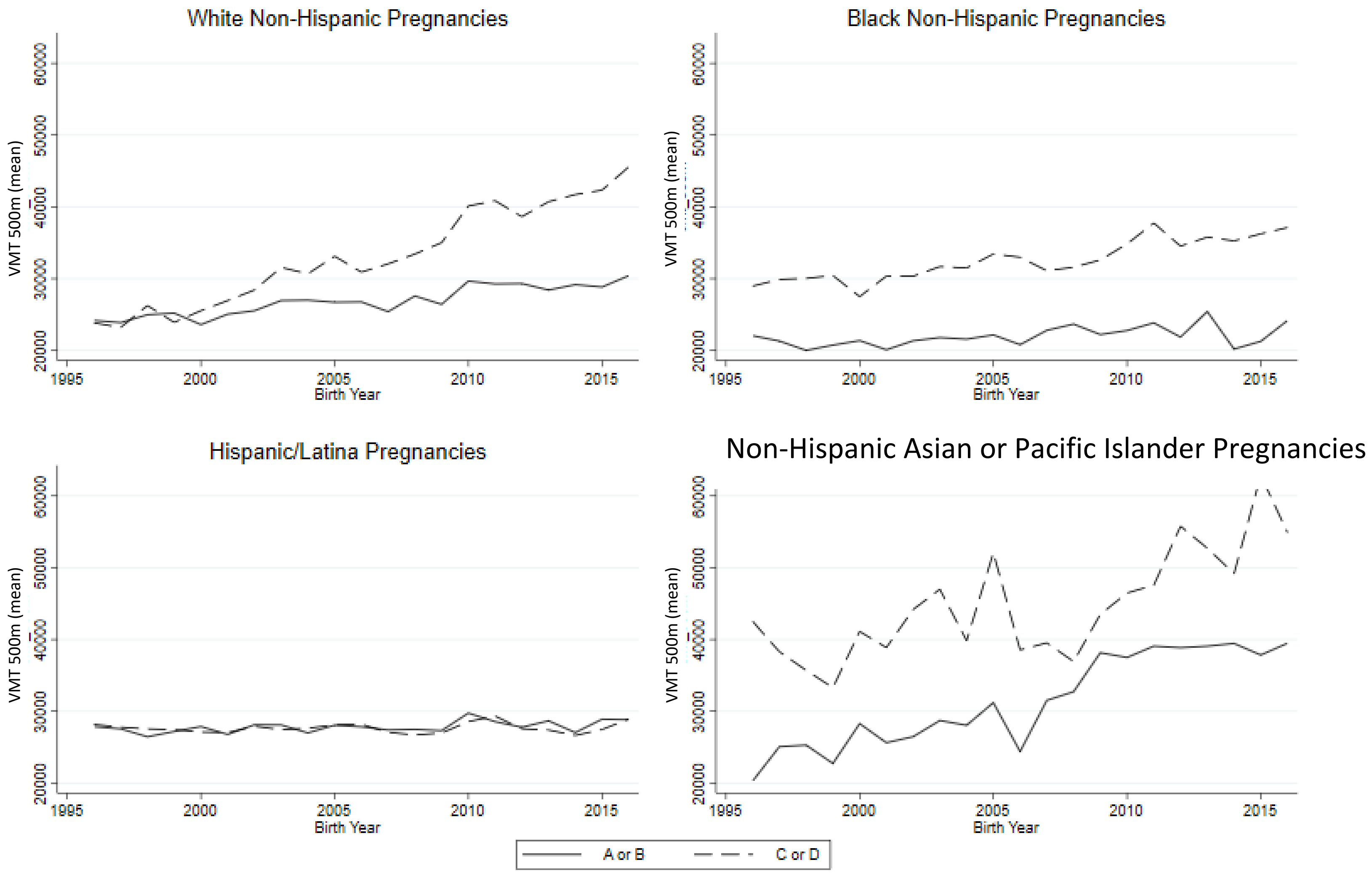


© 2023 Willis MD et al. *JAMA Network Open.*

**Supplemental Figure 16:** Summary of NO2 by individual race/ethnicity and historical neighborhood disinvestment from 1996-2016. A or B: the "least risky" (i.e., not redlined) areas; C or D: the "most risky" (i.e., redlined) areas

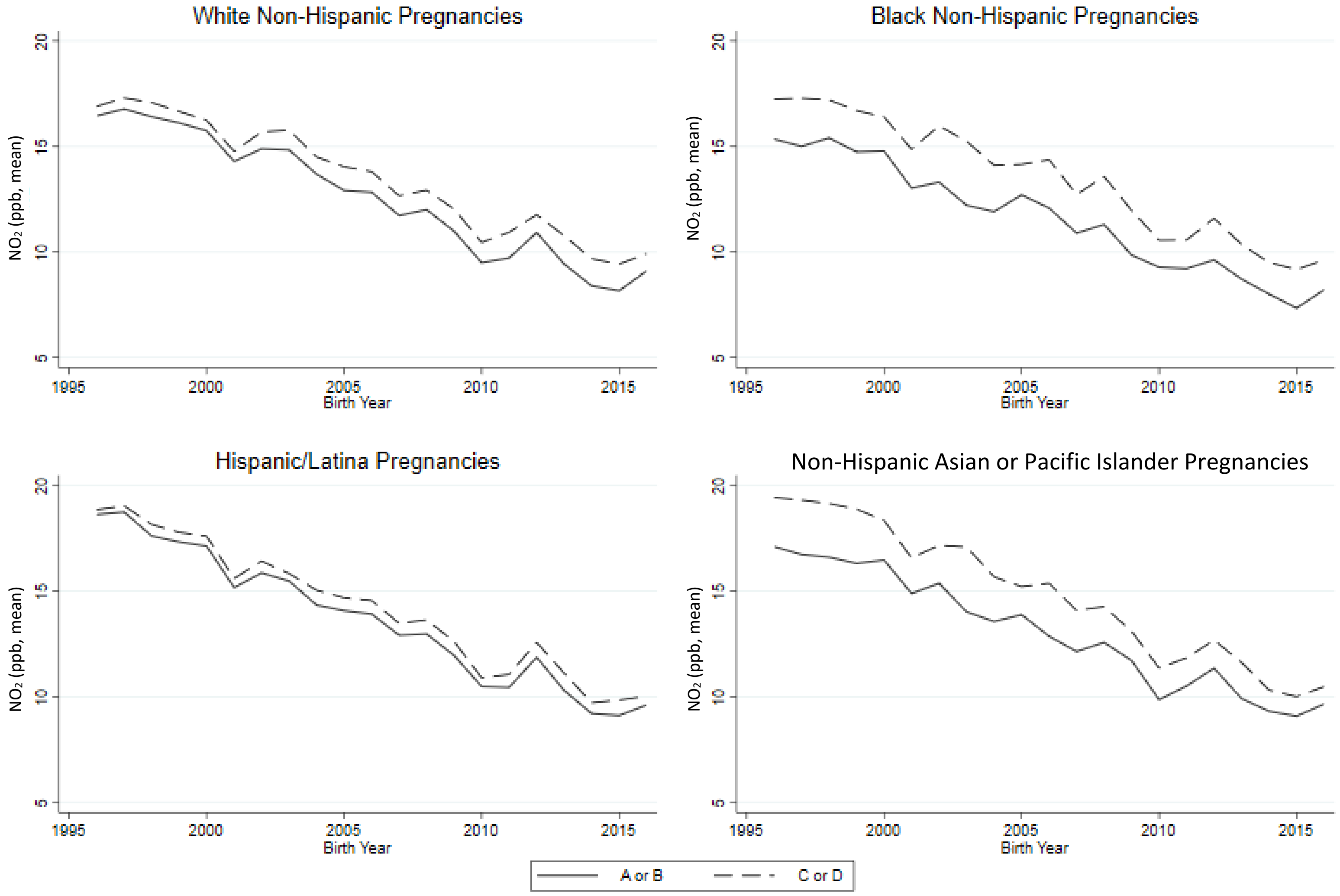


© 2023 Willis MD et al. *JAMA Network Open.*

**Supplemental Figure 17:** Summary of truck VMT 500 m by individual race/ethnicity and historical neighborhood disinvestment from 1996-2016. A or B: the "least risky" (i.e., not redlined) areas; C or D: the "most risky" (i.e., redlined) areas

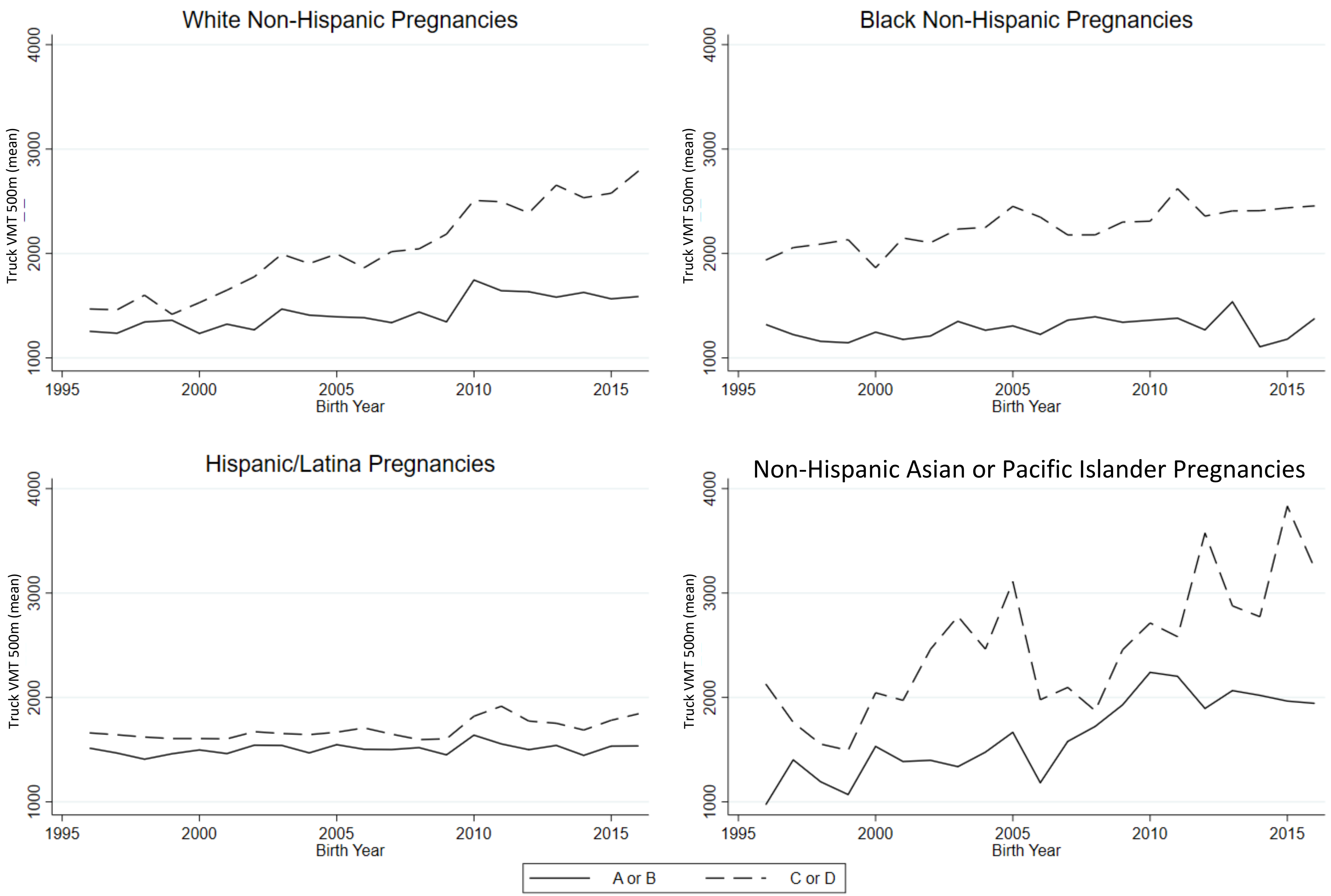


© 2023 Willis MD et al. *JAMA Network Open.*

**Supplemental Figure 18:** Summary of vehicle cancer risk by individual race/ethnicity and historical neighborhood disinvestment from 1996-2016. A or B: the "least risky" (i.e., not redlined) areas; C or D: the "most risky" (i.e., redlined) areas

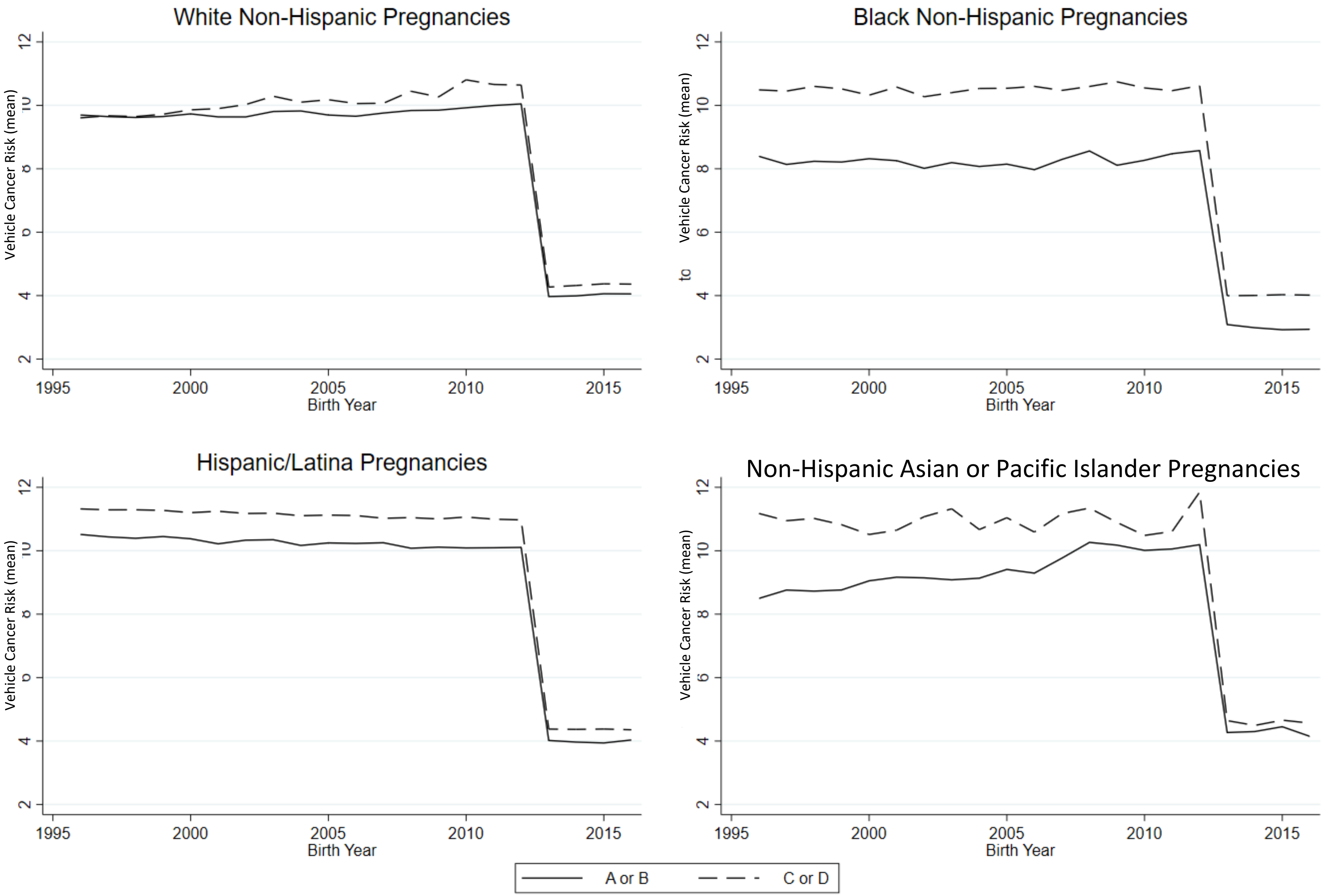


© 2023 Willis MD et al. *JAMA Network Open.*
