# Supplementary material for: Changes in Socioeconomic Disparities for Traffic-Related Air Pollution Exposure During Pregnancy Over a 20-Year Period in Texas
Source: JAMA Netw Open. 2023 Aug 11;6(8):e2328012. doi: 10.1001/jamanetworkopen.2023.28012 (PMC10422188; doi:10.1001/jamanetworkopen.2023.28012)
Supplement: Supplement 2. — Data Sharing Statement [file jamanetwopen-e2328012-s002.pdf]

Data Sharing Statement

Willis. Changes in Socioeconomic Disparities for Traffic-Related Air Pollution Exposure During Pregnancy Over a 20-Year Period in Texas. *JAMA Netw Open*. Published August 11, 2023. doi:10.1001/jamanetworkopen.2023.28012

Data
Data available: No

Additional Information
Explanation for why data not available: Health data can be obtained by reasonable request to the Texas Department of Health and Human Services, Vital Statistics Unit. All other data sources are referenced in the text.
